# Silver Nanoparticles Stabilized with Phosphorus-Containing Heterocyclic Surfactants: Synthesis, Physico-Chemical Properties, and Biological Activity Determination

**DOI:** 10.3390/nano11081883

**Published:** 2021-07-22

**Authors:** Martin Pisárčik, Miloš Lukáč, Josef Jampílek, František Bilka, Andrea Bilková, Ľudmila Pašková, Ferdinand Devínsky, Renáta Horáková, Matěj Březina, Tomáš Opravil

**Affiliations:** 1 Department of Chemical Theory of Drugs, Faculty of Pharmacy, Comenius University, SK-83232 Bratislava, Slovakia; lukac@fpharm.uniba.sk; 2 Department of Analytical Chemistry, Faculty of Natural Sciences, Comenius University, SK-84215 Bratislava, Slovakia; josef.jampilek@uniba.sk; 3 Department of Cell and Molecular Biology of Drugs, Faculty of Pharmacy, Comenius University, SK-83232 Bratislava, Slovakia; bilka@fpharm.uniba.sk (F.B.); bilkova@fpharm.uniba.sk (A.B.); paskova@fpharm.uniba.sk (Ľ.P.); 4 Faculty of Pharmacy, Comenius University, SK-83232 Bratislava, Slovakia; devinsky@fpharm.uniba.sk; 5 Comenius University, SK-81499 Bratislava, Slovakia; renata.horakova@uniba.sk; 6 Materials Research Centre, Faculty of Chemistry, University of Technology, CZ-61200 Brno, Czech Republic; brezina@fch.vut.cz (M.B.); opravil@fch.vutbr.cz (T.O.)

**Keywords:** phosphonium, silver nanoparticles, dynamic light scattering, zeta potential, Hep G2 cells, cytotoxicity

## Abstract

Phosphorus-containing heterocyclic cationic surfactants alkyldimethylphenylphospholium bromides with the alkyl chain length 14 to 18 carbon atoms were used for the stabilization of silver nanodispersions. Zeta potential of silver nanodispersions ranges from +35 to +70 mV, which indicates the formation of stable silver nanoparticles (AgNPs). Long-chain heptadecyl and octadecyl homologs of the surfactants series provided the most intensive stabilizing effect to AgNPs, resulting in high positive zeta potential values and smaller diameter of AgNPs in the range 50–60 nm. A comparison with non-heterocyclic alkyltrimethylphosphonium surfactants of the same alkyl chain length showed better stability and more positive zeta potential values for silver nanodispersions stabilized with heterocyclic phospholium surfactants. Investigations of biological activity of phospholium-capped AgNPs are represented by the studies of antimicrobial activity and cytotoxicity. While cytotoxicity results revealed an increased level of HepG2 cell growth inhibition as compared with the cytotoxicity level of silver-free surfactant solutions, no enhanced antimicrobial action of phospholium-capped AgNPs against microbial pathogens was observed. The comparison of cytotoxicity of AgNPs stabilized with various non-heterocyclic ammonium and phosphonium surfactants shows that AgNPs capped with heterocyclic alkyldimethylphenylphospholium and non-heterocyclic triphenyl-substituted phosphonium surfactants have the highest cytotoxicity among silver nanodispersions stabilized by the series of ammonium and phosphonium surfactants.

## 1. Introduction

Cationic surfactants containing quaternary phosphorus atom in their molecule hydrophilic part are not as popular as their ammonium analogues. Weaker stability of tertiary phosphanes used for their synthesis and generally more complex synthesis may be responsible for their lesser popularity among the family of cationic surfactants. However, they still manage to attract scientific interest due to their unusual properties. They were successfully applied in numerous industrial fields of applications such as iron ore flotation [1], oil recovery [2], electrophoresis [3], corrosion inhibition [4,5], and others. Phosphonium surfactants were successfully used as organic modifiers of clay to prepare poly(ethylene terephthalate) nanocomposites [6]. Due to their higher thermal stability, organoclays containing phosphonium surfactants are more suited to preparing nanocomposites than are quaternary ammonium-modified clays [7,8]. Moreover, phosphonium surfactants provide some unique biological properties in the field of targeted mitochondrial delivery [9]. Stearyltriphenylphosphonium-functionalized liposomes were used to specifically target the mitochondria [10,11]. Like ammonium surfactants, phosphonium surfactants show potent antimicrobial activity when modified by polymers [12]. Ethyltriphenylphosphonium bromide was applied to modify polyacrylonitrile fibers which resulted in good antibacterial performance against *E. coli* and *S. aureus* [13]. Antimicrobially active polymers were prepared by copolymerization of *N*-isopropylacrylamide with methacryloyloxyethyl trialkylphosphonium chloride [14]. Various antimicrobial polymeric phosphonium salts prepared via polymerization showed much higher antibacterial and antifungal activities than the polymeric ammonium surfactant analogues [15,16]. Biocidal performance superior to that of ammonium surfactants was found for phosphonium salts applied as polymer modifiers in the preparation of chitosan [17] and natural rubber [18] utilizing triphenyl- and tributylphosphonium salts. Among the variety of structural modifications of phosphonium surfactants [19], notable attention is paid to the investigations of the application potential of triphenyl-substituted phosphonium surfactants [20,21,22,23,24,25,26,27,28]. The reason for their popularity is a relatively simple and cheap synthesis. Despite the presence of three phenyl rings in their molecular structure, they have good solubility in water, even up to the alkyl chain length of 18 carbon atoms [26,29]. Phosphonium salts with just alkyl substituents are known for their ability to form thermotropic mesophases and ionic liquids [30,31].

Silver nanoparticles (AgNPs) continue to attract broad industrial, scientific, and biomedical interest due to numerous ways and procedures of their preparation [32] and their vast application potential. Despite a limited information toxicity and in vivo biological data of AgNPs, they turned out to be proven antibacterial agents in medical applications [33,34,35]. They were found to have anticancer [36,37,38], antioxidative [39,40], and anti-inflammatory [41,42] effects. Industrial fields of AgNPs applications are mostly represented by cosmetics [43,44], textile coating [45,46], food storage [47], and others. The known instability of silver nanodispersions resulted in a large number of physical studies oriented on the selection of suitable stabilizers of AgNPs. The group of potential stabilizers of silver nanoparticles is considerably wide, ranging from polymers and surfactants to macrocyclic compounds such as calixarenes [48]. Of the broad family of cationic surfactants, a special group of double-chain gemini surfactants with various molecular composition of surfactant spacer turned out to be potent stabilizers of AgNPs [49,50]. Our previous structure-property relationship studies of stabilization potential of bisammonium gemini surfactants [49], single-chain ammonium [51], and phosphonium [52] surfactants revealed the complex effect of various structural features in surfactant molecule on physical properties and biological activity of surfactant-stabilized AgNPs. The investigations within the present work are extended towards the group of phosphorus-containing surface-active molecules featuring a five-membered heterocyclic ring in the molecule hydrophilic part whose synthesis and aggregation properties we reported previously [29]. The obtained experimental data provide the opportunity to extend the knowledge of surfactant molecular structure—biological activity of AgNPs relationship towards heterocyclic amphiphilic molecules, thus contributing to the search for an efficient, stable, and biologically active silver nanodispersion.

## 2. Materials and Methods

### 2.1. Used Surfactants and Synthesis of AgNPs

**Scheme 1:**
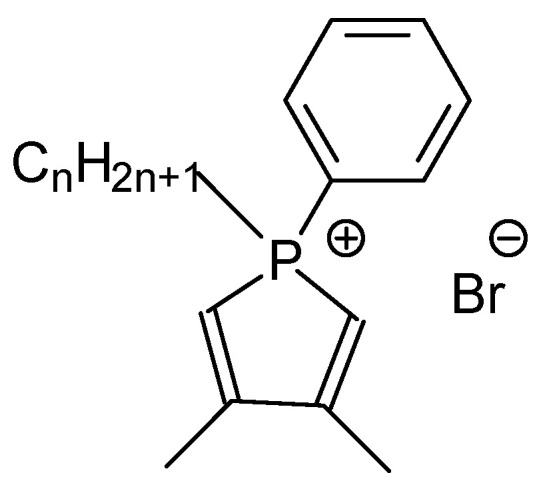
Molecular structure of alkyldimethylphenylphospholium surfactants **14pl** (n = 14), **15pl** (n = 15), **16pl** (n = 16), **17pl** (n = 17), **18pl** (n = 18).

Cationic alkyldimethylphenylphospholium surfactants with an alkyl chain length ranging from 14 to 18 carbon atoms (hereinafter referred to as **14pl**–**18pl**, Scheme 1) were synthesized as described previously including the determination of their surface activity and aggregation properties in aqueous solutions [29].

For the preparation of alkyldimethylphenylpospholium surfactant (**pl**) stabilized AgNPs, we have used the same procedure as described in our previous study [49]. As the published studies indicate, AgNPs are stabilized by the formation of a protective surfactant bilayer composed of single-chain or gemini surfactant molecules. The formation and composition of surfactant-capped AgNPs and their organic shell are mostly documented by the changes in the UV-VIS spectra due to the plasmon resonance effect and changes in nanoparticle surface charge represented by the variation of zeta potential [49,51,52,53,54].

The analysis of physical parameters (spectroscopic properties, hydrodynamic size, and zeta potential) of AgNPs stabilized by the whole series of pl surfactants was carried out three days after the synthesis of AgNPs to ensure equal conditions for the comparison of physical parameters of AgNPs. The formed silver nanodispersions were found to be stable for several months with no signs of phase separation.

The effect of other factors such as temperature on physical properties of AgNPs is rather complex as the non-monotonous temperature dependence of formation and growth of AgNPs is reported [55]. To avoid having temperature as a variable parameter in our experiments, we performed the synthesis and physical and biological investigations of the formed AgNPs at 25 °C to allow to determine the properties of the synthesized AgNPs exclusively as a function of molecular structure of the stabilizing surfactants.

The hydrophobicity level of the molecular structure of stabilizing surfactants turned out to be a significant factor affecting the aging and stability of the formed silver nanodispersions, as well. It results from our previous experiments that in the case of single chain surfactants, the alkyl chain length of 14 carbon atoms and more is sufficient to efficiently stabilize silver nanodispersions. Dodecyl chains in a single chain cationic surfactant molecule like DTAB turned out to be insufficient for the stabilization of AgNPs and the formed Ag nanodispersion decomposed into liquid phase and solid silver phase within several hours after the preparation [51]. On the other hand, double chain gemini bisammonium surfactants with two dodecyl chains provide efficient stabilization of AgNPs [49]. This underlines the importance of sufficient hydrophobicity of stabilizing surfactant molecules as a critical parameter in the process of stabilization of AgNPs.

### 2.2. Spectroscopic Measurements

Absorption spectra in the visible range were carried in the wavelength interval 280–800 nm with a temperature of 25 °C using Genesys 10S equipment (ThermoFisher Scientific, Waltham, MA, USA). Prior to the measurements, silver nanodispersions were diluted to reach measurable absorbance.

### 2.3. Dynamic Light Scattering and Scanning Electron Microscopy

Hydrodynamic size of silver nanoparticles was measured utilizing dynamic light scattering method (Brookhaven BI9000 correlator and 200SM goniometer, argon laser, 514.5 nm wavelength, Brookhaven Instruments Corporation, Holtsville, NY, USA). The intensity time fluctuations of the scattered light were detected at a scattering angle of 90° and a temperature 25 °C. Translation diffusion coefficient was calculated from the fit of time correlation functions utilizing the cumulants method for each surfactant and silver-to-surfactant molar ratio value. Hydrodynamic diameter of AgNPs was calculated from the translation diffusion coefficient using the Stokes–Einstein formula. The mean value and standard deviation of the hydrodynamic size were calculated from 5 repeated measurements. Polydispersity during the nanoparticle size analysis was found to be in the range 0.24–0.33 with the error value 3 to 8% for all investigated Ag/surfactant systems at each silver-to-surfactant molar ratio value. Nanoparticle size distributions were obtained using the CONTIN numerical algorithm applied to the time correlation function. For more details regarding mathematical background of the used data evaluation methods, screenshots of autocorrelation functions, and particle size analysis, see the Supplementary Material.

Field emission gun scanning electron microscope (JEOL JSM-7600F, Tokyo, Japan) was used for the scanning electron microscopy (SEM) images of AgNPs. The accelerating voltage was 1.5 kV, working distance 1.5 mm, and probe current 80 pA. Process of Au sputtering was used to prevent unfavorable sample conductivity.

### 2.4. Zeta Potential Measurements

Zeta potential measurements were performed with the Brookhaven BI ZetaPlus analyzer (Brookhaven Instruments Corporation, Holtsville, NY, USA). Zeta potential values were calculated from the measured electrophoretic mobility of AgNPs at 25 °C using the Smoluchowski equation for charged colloidal particles. The plotted zeta mean values were calculated from the set of 20 repeated measurements. Screenshots of zeta potential peaks obtained from the measurements for all investigated Ag/surfactant systems at each silver-to-surfactant molar ratio value are shown in the Supplementary Material.

### 2.5. Determination of Biological Activities

The broth dilution minimum inhibitory concentration (MIC) method was used for antimicrobial activity evaluation of tested substances/nanoparticles as described in [56]. In experiments, these strains of microorganisms were used: *Escherichia coli* CNCTC 377/79 (Gram-negative bacterium), *Staphylococcus aureus* CNCTC Mau 29/58 (Gram-positive bacterium), and yeast *Candida albicans* CCM 8186. Both bacterial strains were obtained from the Czech National Collection of Type Cultures (Czech Republic) and yeast was purchased from the Czech Collection of Microorganisms (Czech Republic).

Cytotoxic activity of nanoparticles was tested by viability of HepG2 cells (ATCC HB-8065). Cell cultivation conditions are described in detail in [52]. Briefly, cells were seeded at 70% confluency into wells of 24-well culture plates and were incubated for 24 h. Then, the tested substances in increasing concentrations (0–60 μmol/L) were added and cells were incubated for the next 24 h. After the removing of medium and fixation with 50% ethanol, the percentage of the live cells was indicated by Janus Green B staining [57] with modifications described in [52].

## 3. Results and Discussion

### 3.1. UV-VIS Spectra

**Figure 1:**
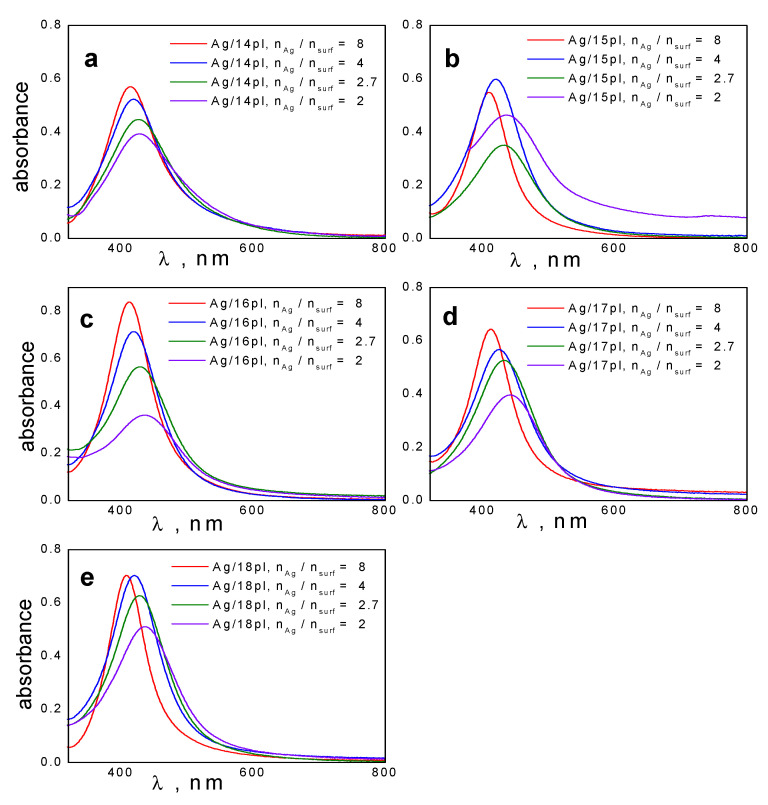
UV-VIS spectra of AgNPs stabilized with alkyldimethylphenylphospholium surfactants for various molar ratio values n_Ag_/n_surf_ of the following silver nanodispersions: (**a**): Ag/**14pl**, (**b**): Ag/**15pl**, (**c**): Ag/**16pl**, (**d**): Ag/**17pl**, (**e**): Ag/**18pl**.

In Figure 1a–e, visible spectra of AgNPs stabilized with alkyldimethylphenylphospholium surfactants of the alkyl chain length 14–18 carbon atoms (**14pl**, **15pl**, **16pl, 17pl,** and **18pl**) are shown. The peaks are found in the interval of wavelengths 408–441 nm because of the presence of the plasmon resonance effect. As the plots indicate, both peak position and maximum absorbance change with varying silver-to-surfactant molar ratio n_Ag_/n_surf_ surf between the values 2 and 8.

**Figure 2:**
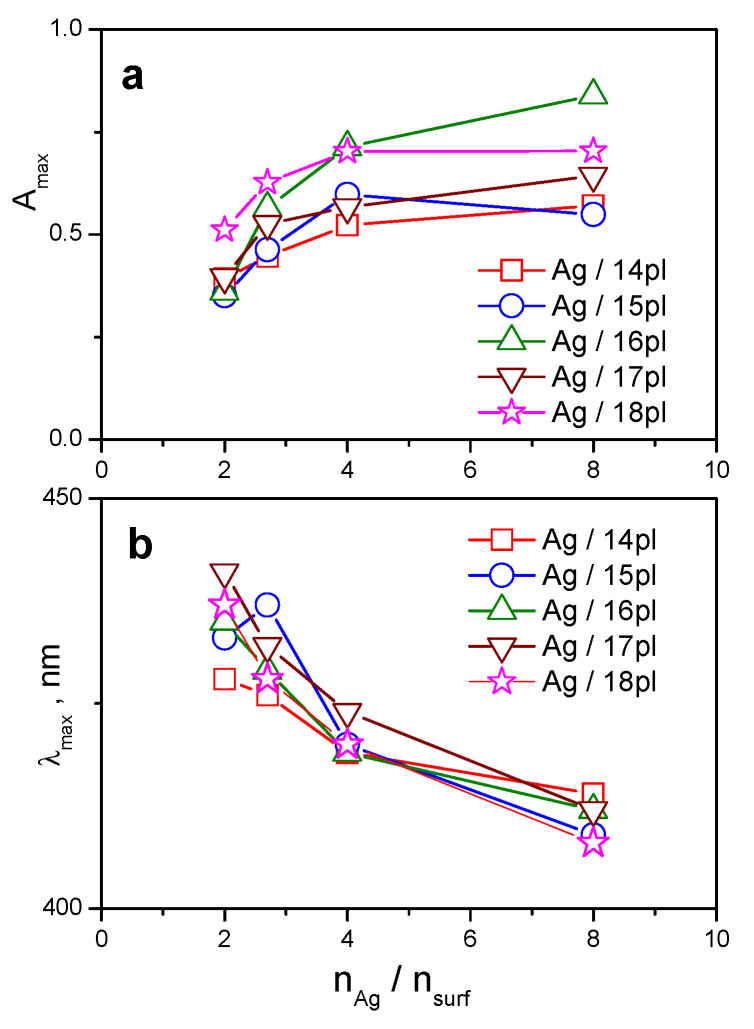
(**a**): Maximum absorbance intensity A_max_ (**b**): maximum absorbance wavelength λ_max_ plotted as a function of n_Ag_/n_surf_.

The changes in maximum absorbance intensity A_max_ and maximum absorbance wavelength λ_max_ vs. the molar ratio n_Ag_/n_surf_ are depicted in Figure 2.

The plot in Figure 2a indicates that A_max_ decreases with the increasing amount of surfactant in silver nanodispersions, i.e., with the decreasing n_Ag_/n_surf_ molar ratio. The absorbance decrease is steeper at smaller n_Ag_/n_surf_ values. It is a consequence of the formation of protective surfactant bilayer surrounding silver nanoparticle. Figure 2a shows that the differences in surfactant alkyl chain length do not affect A_max_ at a very small n_Ag_/n_surf_ molar ratio (the data points overlap in the plot). Moreover, the A_max_ decrease at small n_Ag_/n_surf_ is more pronounced for long-chain surfactant members of the series **16pl**, **17pl**, and **18pl**, thus indicating the importance of alkyl chain length in the formation of protective surfactant bilayer and nanoparticle stabilization. The λ_max_ curves vs. n_Ag_/n_surf_ for all investigated surfactant alkyl lengths (Figure 2b) indicate red shift of maximum absorbance wavelength with the increasing amount of surfactant in silver nanodispersion. The red shift in visible spectra was also observed in case of AgNPs stabilized with single chain cationic cetyltrimethylammonium bromide surfactant and was related to an increase in the solvent refractive index [58].

### 3.2. Nanoparticle Size and Zeta Potential

**Figure 3:**
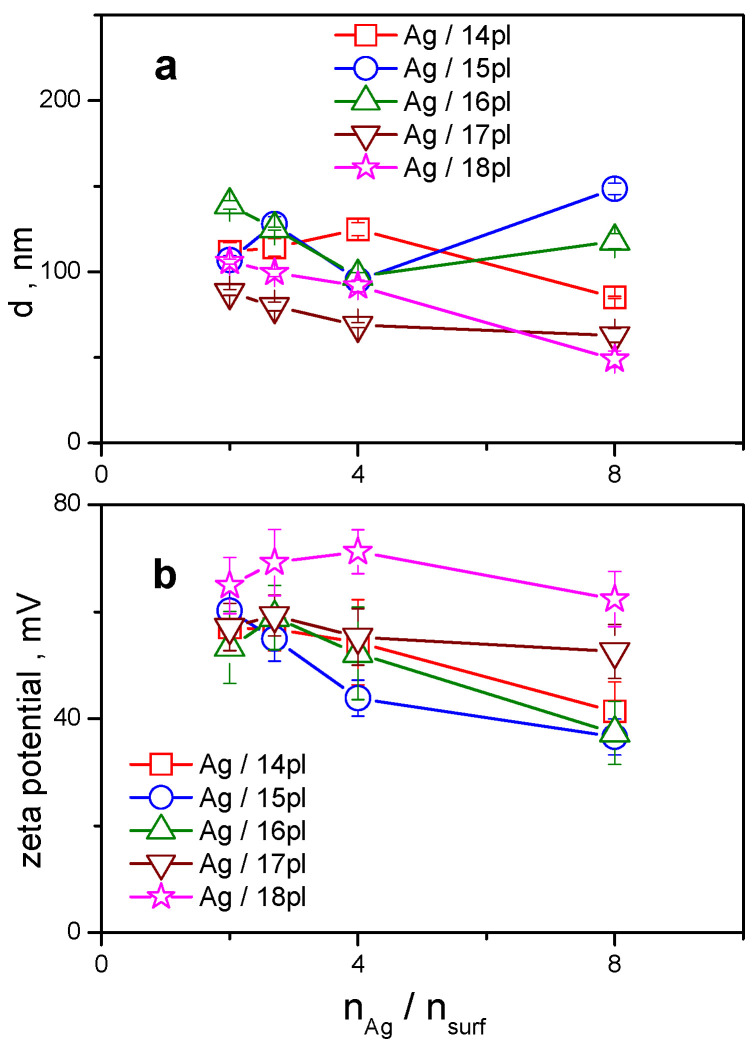
(**a**): Hydrodynamic size d of silver nanoparticles stabilized with phospholium surfactants **14pl**–**18pl** plotted against n_Ag_/n_surf_. (**b**): zeta potential plotted against n_Ag_/n_surf_.

The mean size of silver nanoparticles stabilized with phospholium surfactants with the alkyl chain length 14 to 18 carbon atoms is plotted against n_Ag_/n_surf_ (Figure 3a). The data were obtained from dynamic light scattering (DLS) measurements. Plots of autocorrelation curves and the data analysis of related physical quantities performed by the DLS equipment control software as well as the plots of zeta potential peaks are shown in the Supplementary Material.

Hydrodynamic size of AgNPs stabilized with phospholium surfactants varies in the range 49–149 nm. For all investigated surfactants, the size of AgNPs increases with increasing the amount of surfactant in nanodispersion (right-to-left direction in the plot) below n_Ag_/n_surf_ = 4. This indicates the formation of protective surfactant bilayer surrounding the nanoparticle. The dependence of AgNPs size on the alkyl chain length of phospholium surfactants results from the plot in Figure 3a, as well. This dependence is most obvious at the largest silver-to-surfactant molar ratio value and for long-chain homologs **17pl** and **18pl** that provide intense stabilizing effect to AgNPs even at the lowest amount of surfactant in Ag nanodispersion. The size of Ag/**17pl** and Ag/**18pl** nanoparticles is still well below 70 nm. The size of AgNPs stabilized with **pl** surfactants of a shorter alkyl chain length (14, 15, 16 carbon atoms) is significantly larger at this molar ratio. This indicates the importance of alkyl chain length factor in the process of the formation of stable AgNPs with a small size. When increasing surfactant amount in Ag nanodispersion, the AgNP size moderately increases. At small n_Ag_/n_surf_ values, the differences in AgNP size disappear and the AgNP size seems to converge to the size values somewhere between 100–110 nm. The insufficient alkyl chain length that produces less stable AgNPs with large size, is compensated to a certain extent by increasing the amount of short-chain surfactant in Ag nanodispersion.

A similar trend to that observed for the AgNP hydrodynamic size dependence on n_Ag_/n_surf_ molar ratio is also observed for the zeta potential changes as a function of the silver-to-surfactant molar ratio (Figure 3b). Zeta potential of AgNPs stabilized by all investigated surfactants is found to be positive enough (above +40 mV) which indicates good stabilizing effect of phospholium surfactants on AgNPs. Similarly, zeta potential increases with the increasing surfactant amount which is to relate to the increased stability of Ag nanodispersions because of the presence of surfactant molecules. The largest stabilizing effect on AgNPs is attained by the long-chain surfactant **18pl** with high positive zeta values between +64 and +72 mV in the whole range of molar ratio values. The zeta values as a function of surfactant alkyl chain length are more scattered at large n_Ag_/n_surf_ values. The dependence on the alkyl chain length is diminished as n_Ag_/n_surf_ decreases towards small values, thus making nanodispersions more stable by the presence of larger surfactant amount. This effect is like that observed in Figure 3a.

**Figure 4:**
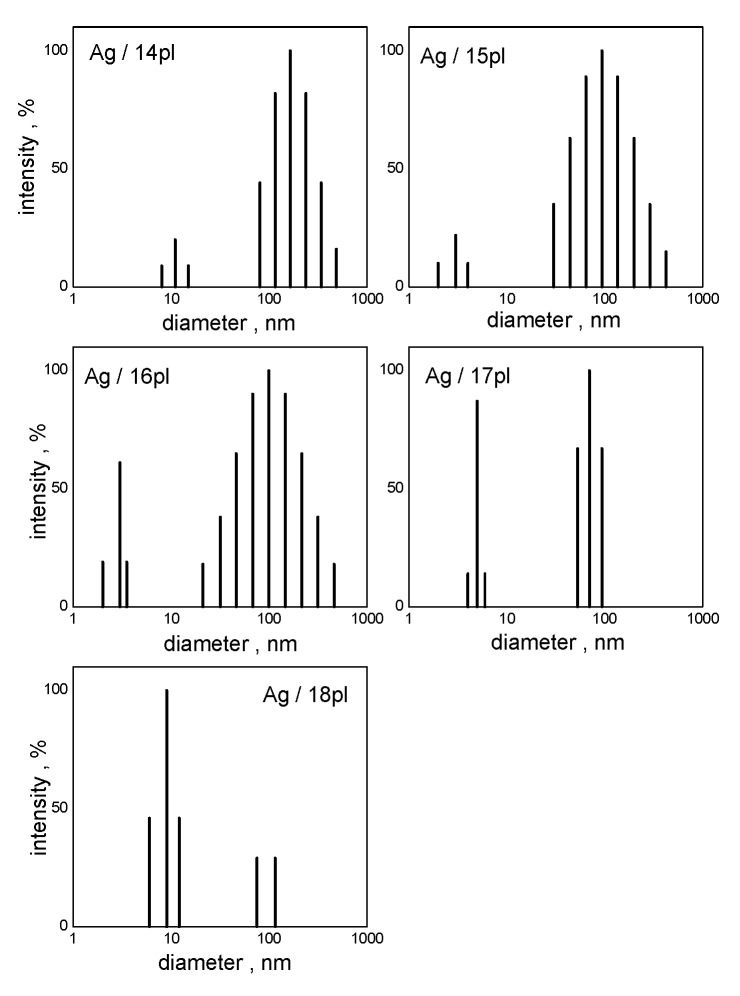
Particle size spectra of AgNPs stabilized with phospholium surfactants at n_Ag_/n_surf_ = 4.

Particle size spectra (Figure 4) determined at n_Ag_/n_surf_ = 4 show AgNPs size distributions for stabilizing surfactants **14pl**, **15pl**, **16pl**, **17pl**, and **18pl**.

The bimodal particle size distribution shows the population of small nanoparticles of the diameter 5–10 nm (Figure 4). This population of small nanoparticles seems to be increasing in terms of intensity with the increasing alkyl chain length. The peak in the particle size spectra related to the particles approx. 100 nm large in size may be related to the population of individual large nanoparticles or clusters of smaller nanoparticles. According to the published studies, chemical method of AgNPs preparation utilizing reducing agents such as sodium borohydride, hydrazine, sodium citrate, and others, results in the formation of mostly spherical silver nanoparticles that are larger in size, typically in the size range 40–60 nm [59]. This size polydispersity of AgNPs corresponds with the broad peaks in particle size spectra (Figure 4), especially for AgNPs stabilized with short-chain surfactants **14pl**–**16pl**. On the other hand, worse stabilizing effect of short-chain surfactants may result in the formation of AgNPs prone to agglomerate into larger clusters, which would explain the presence of the peaks around the size of 100 nm in the particles size spectra.

**Figure 5:**
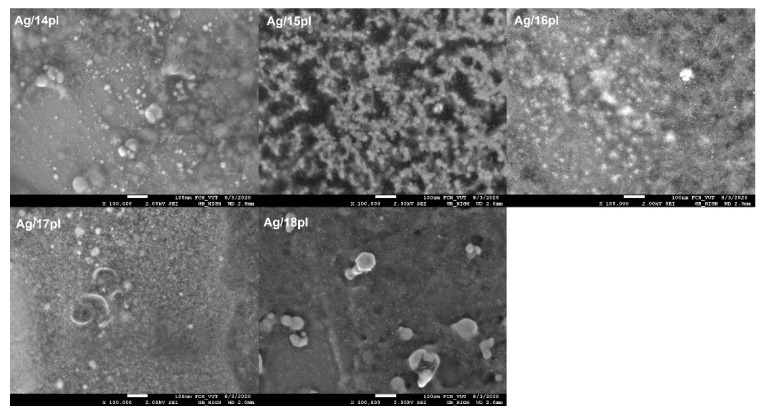
SEM images of AgNPs stabilized with phospholium surfactants.

The SEM images of AgNPs (Figure 5) indicate the coexistence of small AgNPs with large aggregates. High resolutions SEM images are shown in the Supplementary material. The trend of increasing population of small AgNPs with the increasing alkyl chain length of stabilizing surfactant (Figure 5) corresponds with that shown in particle size distributions in Figure 4.

The long-chain phospholium surfactants have the tendency to stabilize Ag nanodispersions more efficiently than the short-chain ones which results in the nanoparticles size decrease in favor of the formation of small nanoparticles. This observation seems also to interplay with the mean AgNP size obtained from light scattering experiments and with the zeta potential values where a significant decrease in mean nanoparticle size and increase in stability represented by high positive zeta potential values were found for AgNPs stabilized with long-chain phospholium surfactants **17pl** and **18pl** (Figure 3).

### 3.3. Comparison with Non-Heterocyclic Phosphonium Surfactants

**Figure 6:**
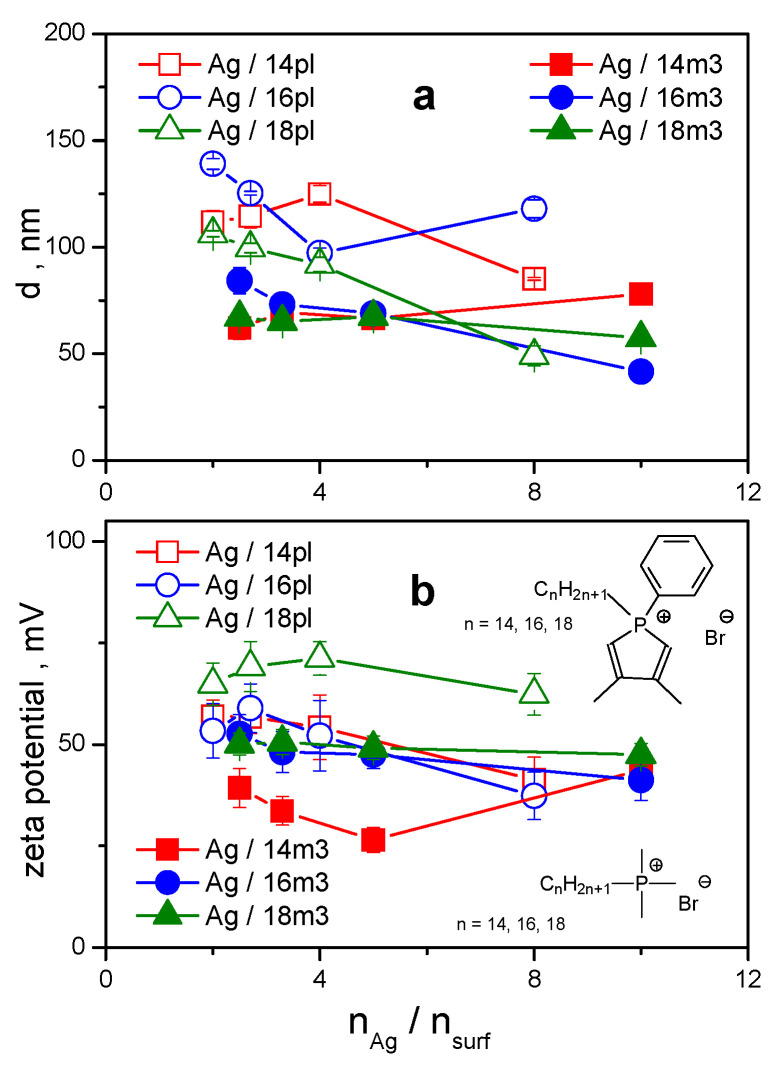
(**a**): Hydrodynamic size d of silver nanoparticles stabilized with heterocyclic alkylphenyldimethylphospholium surfactants **14pl**, **16pl**, **18pl**, and non-heterocyclic alkyltrimethylphosphonium bromides **14m3**, **16m3**, **18m3** [52] plotted against n_Ag_/n_surf_. (**b**): zeta potential of AgNPs plotted against n_Ag_/n_surf_.

The results above indicate the predominant effect of alkyl chain length of phospholium surfactants on size and stability of AgNP. As results from our previous study of AgNPs stabilized with another group of phosphorus-based non-heterocyclic surfactants (phosphonium surfactants) [52], the structure and properties of the polar hydrophilic part of surfactant molecules play an important role in the stabilization and structure of AgNPs as well. Figure 6 shows the comparison of mean size and zeta potential of AgNPs stabilized with heterocyclic alkylphenyldimethylphospholium surfactants **14pl**, **16pl**, **18pl** (open symbols) with our previously published data for non-heterocyclic alkyltrimethylphosphonium bromides (solid symbols, labelled as **14m3**, **16m3**, **18m3**) [52]. Three alkyl chain lengths of 14, 16, and 18 carbon atoms were selected for the comparison.

**Figure 7:**
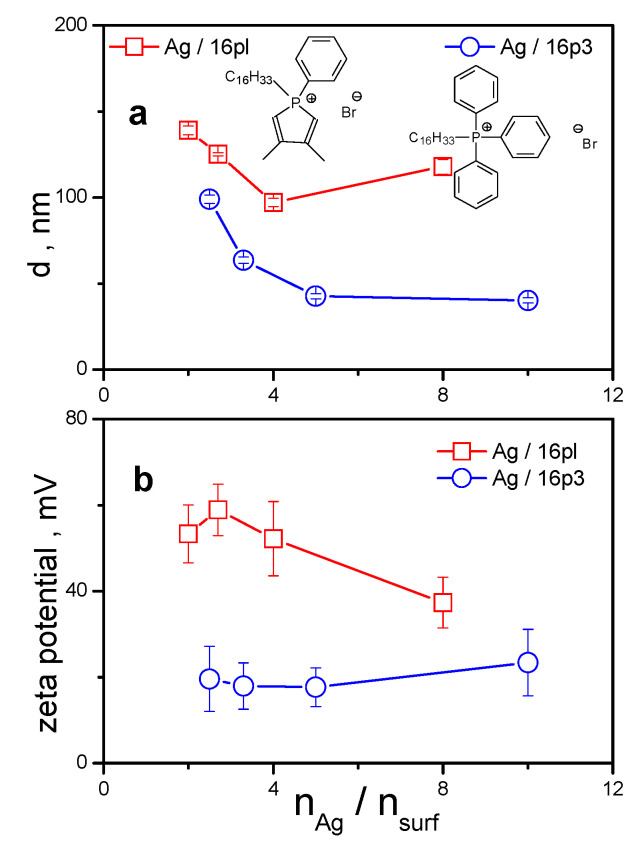
(**a**): Hydrodynamic size d of silver nanoparticles stabilized with hexadecyldimethylphenylphospholium surfactant **16pl**, and with hexadecyltriphenylphosphonium surfactant **16p3** plotted against n_Ag_/n_surf_. (**b**): Zeta potential of AgNPs plotted against n_Ag_/n_surf_.

As can be seen in Figure 6a, the size of AgNPs stabilized with **14pl**, **16pl**, **18pl** is larger than that of AgNPs stabilized with quaternary phosphonium surfactants **14m3**, **16m3**, **18m3**. This observation may be related to the voluminous polar part of phospholium surfactant molecules that is composed of a five-membered heterocyclic ring containing a phosphorus atom and a phenyl substituent. On the other hand, **14m3**, **16m3**, **18m3** surfactants have quaternary phosphorus atom with three small methyl substituents in the polar part of surfactant molecule. The results above indicate that the thickness of surfactant bilayer surrounding AgNP may be affected not only by the length of the alkyl chain but also by the composition of the polar headgroup of surfactant molecule. Results from our previous study concerning stabilization of AgNPs by alkylphosphonium surfactants with various combinations of phenyl and methyl substituents showed that the densest arrangement of surfactant molecules in protective surfactant bilayer surrounding AgNP was attained with alkyltriphenylphosphonium surfactant molecules. The aromatic character, symmetrical composition of triphenyl headgroup and the resulting π-π interactions between neighboring surfactant molecules resulted in their dense arrangement in the bilayer and in nanoparticle size decrease [52]. However, the character of five-membered heterocyclic ring of phospholium headgroup parts of **14pl**, **16pl**, **18pl** is predominantly non-aromatic. Generally, phosphol is a very weakly aromatic, almost non-aromatic substance [60]. Because of this, it can be quaternized to a phospholium cation through the protonation of phosphorus lone electron pair, which results in an antiaromatic character of the whole five-membered heterocyclic ring [61]. As opposed to phosphol, quaternization of, e.g., aromatic pyrrole, does not work for this reason. The absence of π-π interactions and of the resulting sandwich-like arrangement of surfactant molecules containing non-aromatic phospholium heterocycles results in a less than perfect arrangement of surfactant headgroups. Consequently, the hydrodynamic diameter of AgNPs increases. The comparison of zeta values of AgNPs stabilized with **14pl**, **16pl**, **18pl** and **14m3**, **16m3**, **18m3** surfactants (Figure 6b) shows that the determined zeta values do not significantly differ from each other, as the curves and data error bars for individual surfactants zeta values overlap. The two exceptions are Ag/**14m3** nanodispersion with low zeta values due to the found weaker nanodispersion stability [52] and the Ag/**18pl** nanodispersion showing high zeta values around +70 mV. This high stability Ag/**18pl** nanodispersion is not trivial and cannot be simply attributed to the octadecyl chain length, as the Ag/**18m3** nanodispersion stabilized with the phosphonium surfactant of identical alkyl length does not produce such high zeta values. AgNPs stabilized by phosphonium surfactants with triphenyl substituents turned out to have the smallest size among AgNPs stabilized by phosphonium surfactants with other substituents [52]. Surprisingly, zeta potential of alkyltriphenylphosphonium surfactant stabilized AgNPs is rather low when compared with other Ag nanodispersions. We have found that the zeta potential of AgNPs capped with phosphonium surfactants featuring different number of phenyl substituents in surfactant headgroup part decreases with the increasing number of phenyls [52]. Three phenyl groups of triphenyl-substituted phosphonium surfactant can shield the positive charge of phosphonium headgroups through their high electron density [62,63]. As a consequence, effective decrease of positive charge on the surface of silver nanoparticle and decrease in zeta potential value are observed. As an example, one can compare the properties of AgNPs stabilized with hexadecyldimethylphenylphospholium surfactant **16pl** and with hexadecyltriphenylphosphonium surfactants **16p3**. The following plot shows the differences in AgNP size (Figure 7a) and zeta potential (Figure 7b) as a function of silver-to-surfactant molar ratio for AgNPs stabilized by both surfactants. It is obvious from the plot that the dense sandwich-like arrangement of **16p3** triphenyl substituents in Ag nanoparticle organic shell results in low nanoparticle size (Figure 7a). However, the zeta potential of Ag/**16p3** nanodispersions is rather low due the above-described shielding effect of three phenyl substituents when compared with high positive potential of Ag nanodispersions stabilized with non-aromatic **16pl** phospholium surfactant (Figure 7b).

### 3.4. Antimicrobial Activity

**Figure 8:**
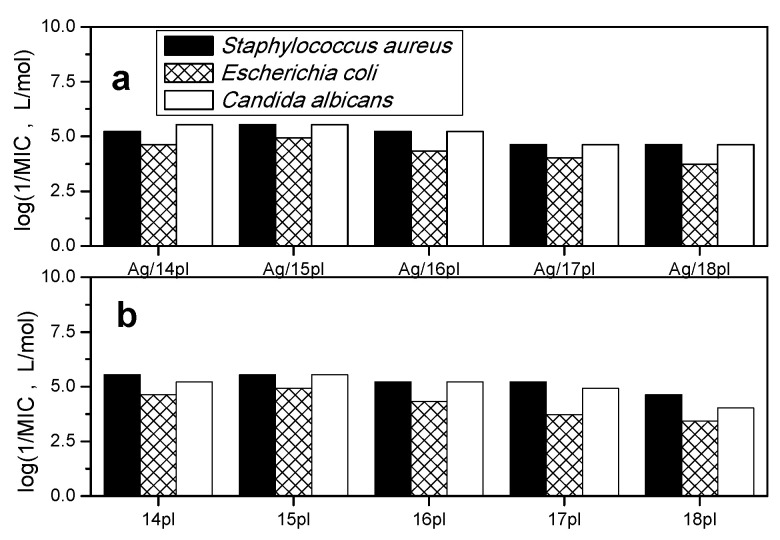
Plot of logarithm of inverse minimum inhibitory concentration log(1/MIC) determined against Gram-positive, Gram-negative bacteria, and a yeast pathogen for (**a**): AgNPs nanodispersions stabilized with alkyldimethylphenylphospholium bromides and (**b**): for aqueous solutions of these surfactants.

Antimicrobial activity of AgNPs stabilized with alkyldimethylphenylphospholium surfactants (Figure 8a) and of surfactant aqueous solutions without the presence of silver (Figure 8b) was determined against Gram-positive, Gram-negative bacteria, and a yeast pathogen. Bar graphs show minimum inhibitory concentration (MIC) plotted as logarithm of its inverse value for individual Ag nanoparticles. The larger log (1/MIC) values, the higher antimicrobial activity.

The plot in Figure 8 indicates that antimicrobial activity of phospholium surfactants is not significantly affected by the presence of silver in the form of nanoparticles. The bar diagrams in Figure 8 show approximately the same antimicrobial activity for both aqueous surfactant solutions and Ag nanodispersions stabilized with alkylphenyldimethylphospholium surfactants at each specific surfactant alkyl chain length. The cut-off effect is known as the nonlinear dependence of biological activity on surfactant molecular parameter such as alkyl chain length [64]. In our case, it is a maximum in the dependence of antimicrobial activity represented by log(1/MIC) values vs. alkyl chain length that is observed at the alkyl length of 15 carbon atoms (Figure 8b). The biological activity of phospholium surfactants in the form of AgNPs shows the same type of dependence on alkyl chain length (Figure 8a). One can conclude that the presence of AgNPs does not dramatically affect the biological action of phospholium surfactants, as the cut-off effect remains present also in antimicrobial activity of Ag nanodispersions. A slight improvement of antimicrobial properties of phospholium surfactants being a part of AgNPs can be observed for long-chain homologs **17pl** and **18pl** of this surfactant series against Gram-negative pathogen *E. coli*. In this case, higher activity than that of surfactant solutions without silver presence is observed (Figure 8a). This situation is different to the antimicrobial action of AgNPs stabilized with alkyltrimethylphoshonium surfactants series Ag/**14m3** to Ag/**18m3** [52]. In that case, a significant improvement of antimicrobial action upon the application of surfactants in the form of AgNPs was observed, especially for long-chain homologs in the surfactant series. We related these changes to the synergic effect of joint antimicrobial action of silver and cationic phosphonium surfactants. No such synergism, however, has been observed for antimicrobial activity of AgNPs stabilized with phospholium surfactants. This may be related to the much bulkier size of phospholium-capped AgNPs that makes the interaction with biological membranes more difficult and hinders the direct interaction of silver atoms with membranes, thus effectively diminishing the contribution of silver to the overall antimicrobial activity of surfactant-modified AgNPs.

### 3.5. Cytotoxicity

**Figure 9:**
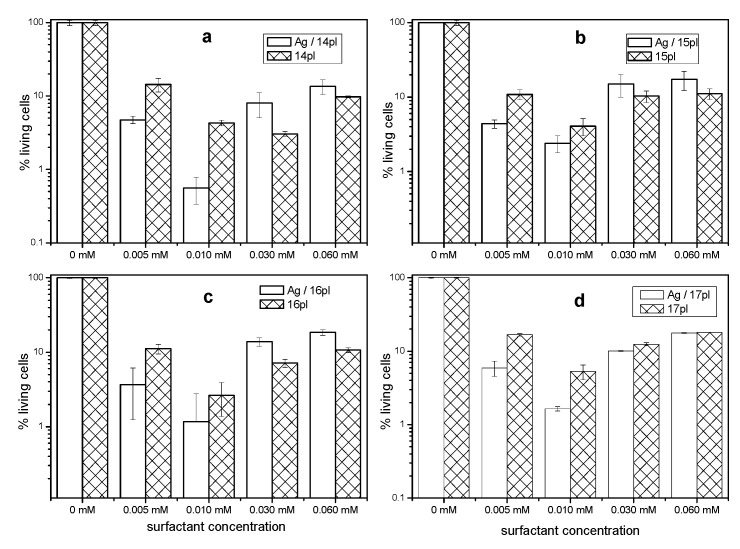
Cytotoxicity of alkylidimethylphenylphospholium surfactant-stabilized silver nanoparticles (**a**: Ag/**14pl**, **b**: Ag/**15pl**, **c**: Ag/**16pl**, and **d**: Ag/**17pl**) and of silver-free surfactant solutions (**a**: **14pl**, **b**: **15pl**, **c**: **16pl**, and **d**: **17pl**) at four different surfactant concentrations. Cell viability of 100% represents no cytotoxic effect on cancer cells.

**Figure 10:**
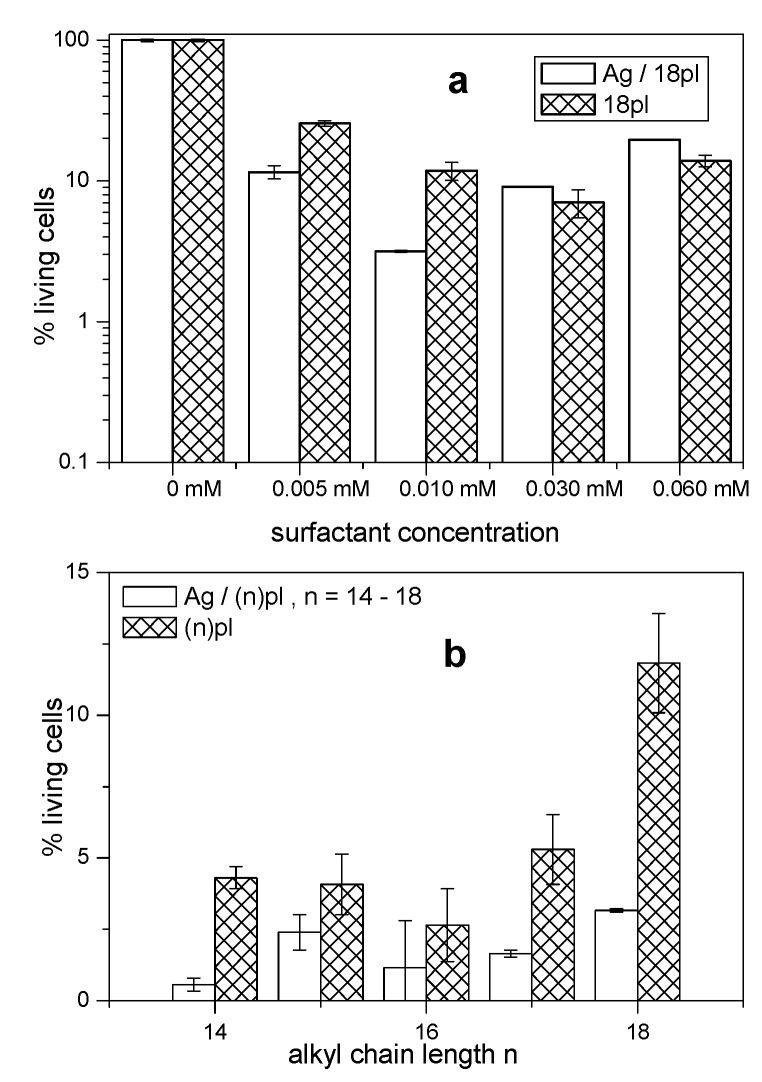
(**a**): Cytotoxicity of Ag/**18pl** nanodispersion and **18pl** surfactant solution. (**b**): Cytotoxicity plotted against alkyl chain length represented by the number of carbon atoms in hydrocarbon chain at the surfactant concentration 0.01 mmol/L.

The bar charts in Figure 9a–d and Figure 10a show the percentage of surviving human carcinoma HepG2 cells plotted against surfactant concentration for AgNPs stabilized with all investigated phospholium surfactants, as well as for silver-free surfactants solutions determined at four different surfactant concentrations. Figure 10b shows the dependence of both series on the alkyl chain length.

**Figure 11:**
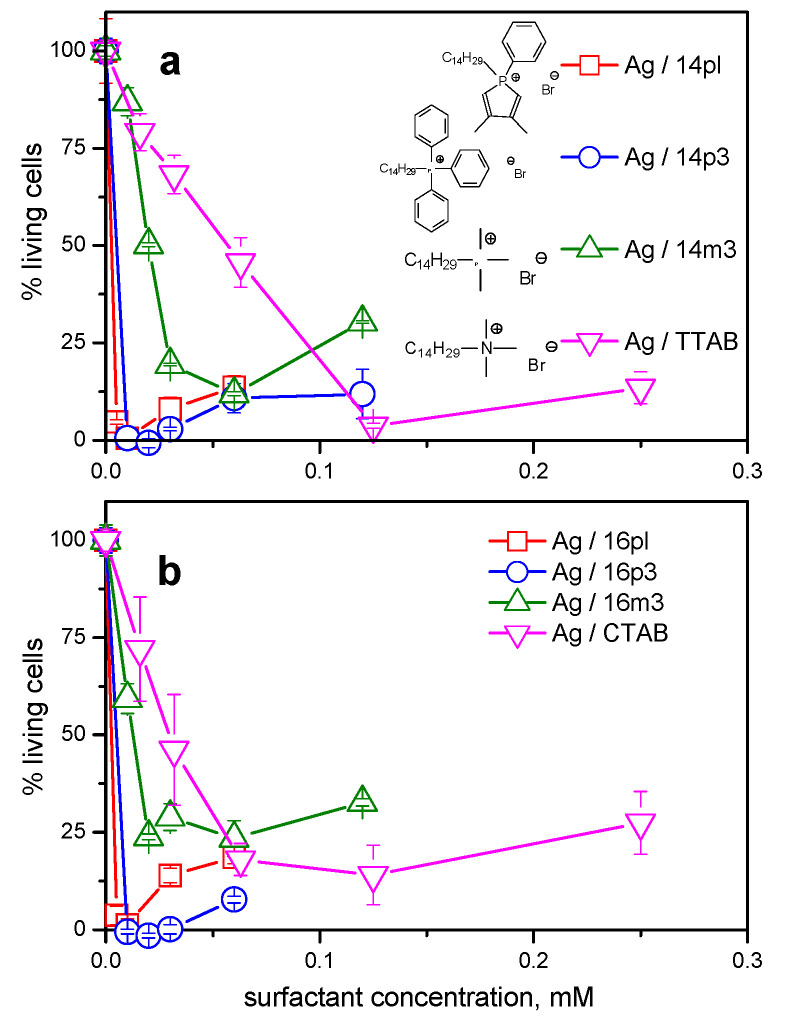
Effect of surfactant headgroup composition on cytotoxic activity at the fixed alkyl chain length. Cytotoxicity vs. surfactant concentration plots of AgNPs stabilized with alkyldimethylphenylphospholium (**pl**), alkyltriphenylphosphonium (**p3**), alkyltrimethylphosphonium (**m3**) and alkyltrimethylammonium (TTAB and CTAB) surfactants. The alkyl chain length (**a**): C_14_ (**b**): C_16_.

In Figure 9a–d and Figure 10a, decrease of viable cells percentage with increasing surfactant concentration is observed up to the concentration 0.01 mmol/L with subsequent increase at higher concentrations. This cell viability increase at higher concentrations is similar to that observed in our previous study [51]. Although the origin of this effect in not quite clear, it may represent a saturation of cytotoxic agent within the interaction with cancer cells and was reported in a few cases in the literature [65,66], as well. In contrast to the antimicrobial activity results (Figure 8), cytotoxicity of AgNPs stabilized with phospholium surfactants is higher than that of silver-free surfactant solutions up to the surfactant concentration 0.01 mol/L. Cytotoxicity of surfactant stabilized AgNPs and of silver-free aqueous surfactant solutions determined at the most efficient surfactant concentration 0.01 mmol/L and plotted as a function of alkyl chain length (Figure 10b) indicates that surfactant stabilized AgNPs are cytotoxically more effective than surfactant solutions. This observation may be related to two structural features that obviously result from the molecular structure of phospholium surfactants: the presence of heterocyclic ring and a phenyl substituent. To shed some light on this issue and evaluate the effect of different surfactant headgroup structure on cytotoxic activity at the fixed alkyl chain length, cytotoxicity vs. surfactant concentration plots are constructed for AgNPs stabilized with alkyldimethylphenylphospholium (**pl**), alkyltriphenylphosphonium (**p3**), alkyltrimethylphosphonium (**m3**), and alkyltrimethylammonium (TTAB and CTAB) surfactants at the alkyl chain length C_14_ (Figure 11a) and C_16_ (Figure 11b). The data for **p3**, **m3**, TTAB, and CTAB surfactants were taken from our previous studies [51,52].

The plots in Figure 11 indicate that AgNPs capped by **pl** and **p3** surfactants are more active than AgNPs stabilized with trimethyl-substituted ammonium or phosphonium surfactants (**m3**, TTAB, CTAB), which is documented by the minimum percentage of surviving cancer cells attained at the lowest surfactant concentration among the investigated surfactants. This underlines the active role of phenyl substituents and heterocyclic phospholium ring in the increased cytotoxicity of AgNPs. Significant increase in cytotoxicity of AgNPs was found with the increasing number of phenyl substituents in phosphonium surfactant headgroup when the percentage of viable cells was found to be well below 1% for triphenyl-substituted phosphonium surfactants [52]. Another observation resulting from the plots is related to the fact that the cytotoxicity curves for Ag/**14pl** and Ag/**14p3** almost overlap at small surfactant concentrations, while cytotoxic activity of Ag/**16p3** is only slightly higher at larger surfactant concentration values (Figure 11). These findings emphasize high cytotoxicity of AgNPs stabilized with non-aromatic heterocyclic phosphorus-based surfactants. The reported studies in the literature indicate that compounds containing five-member heterocyclic moieties such as thiadiazole and thiadiazolium show promising potential as potential antitumor agents due to their ability to disrupt processes related to DNA replication in cancer cells [67] and these compounds were found to be effective against human melanoma cells [68]. Considering the facts above and the strong cytotoxicity of Ag/**pl** nanodispersions, we assume that simultaneous presence of five-membered heterocyclic ring and a phenyl substituent on this ring in the headgroup structure of **pl** surfactants results in an enhanced cytotoxic effect of AgNPs stabilized with alkyldimethylphenylphospholium surfactants.

## 4. Conclusions

Cationic surfactants containing quaternary phosphorus atom within a five-membered heterocyclic ring in the surfactant headgroup part turned out to be potent stabilizers of AgNPs with significant cytotoxic activity. As the present study indicates, positively charged AgNPs were formed and stabilized by alkyldimethylphenylphospholium surfactants in the whole region of investigated alkyl chain length ranging from 14 to 18 carbon atoms. The existence of stable silver nanodispersions was confirmed spectroscopically. In the UV-VIS spectra, plasmon resonance peaks were present for all investigated nanodispersions and their intensity decreased with the increasing surfactant amount in nanodispersion. Particle size and zeta potential analysis revealed that phospholium surfactant-stabilized silver nanoparticles show high positive zeta potential and small size, if surfactant alkyl chain is extended. This is to relate to a better stabilizing efficiency of long-chain surfactants. The comparison of stabilizing effect of aromatic alkyltriphenylphosphonium surfactants with alkyltrimethylphosphonium surfactants indicates that the presence or absence of π-π interactions between phenyl rings in the surfactant headgroup part significantly affects nanoparticle size and zeta potential.

The determination of biological activity of AgNPs stabilized with phospholium surfactants represented by the investigations of cytotoxicity on human carcinoma cell line and antimicrobial activity against bacterial strains provides different outcomes. While the antimicrobial action of AgNPs against Gram-positive, Gram-negative pathogens, and a yeast did not show significant activity improvement over the level of silver-free aqueous surfactant solutions, cytotoxicity of phospholium-stabilized AgNPs was found to be significantly higher than that of surfactant solutions without silver presence. This observation may be related to the presence of aromatic and heterocyclic groups in the molecular structure of surfactants stabilizing AgNPs.

Nanodispersions of AgNPs capped with alkyldimethylphenylphospholium surfactants were found to be stable over long period of time and showed high level of cytotoxicity against cancer cells. This represents a promising contribution to the search for an efficient tool to treat oncologic diseases utilizing nanocarriers that are able to pass biological barriers and directly deliver therapeutic agents to the target site.

## Data Availability

Not applicable.

## Supplementary Materials

The following are available online at https://www.mdpi.com/article/10.3390/nano11081883/s1, 1. Dynamic light scattering measurements and data analysis, 2. Zeta potential measurements, 3. SEM high resolution images.
