# Supplementary material for: Silver Nanoparticles Stabilized with Phosphorus-Containing Heterocyclic Surfactants: Synthesis, Physico-Chemical Properties, and Biological Activity Determination
Source: Nanomaterials (Basel). 2021 Jul 22;11(8):1883. doi: 10.3390/nano11081883 (PMC8399434; doi:10.3390/nano11081883)

Supplementary Material

Silver Nanoparticles Stabilized with Phosphorus-Containing Heterocyclic Surfactants: Synthesis, Physico-Chemical Properties, and Biological Activity Determination

Martin Pisárčik [1,*], Miloš Lukáč [1], Josef Jampílek [2], František Bilka [3], Andrea Bilková [3], Ľudmila Pašková [3], Ferdinand Devínsky [4], Renáta Horáková [5], Matěj Březina [6] and Tomáš Opravil [6]

[1] Department of Chemical Theory of Drugs, Faculty of Pharmacy, Comenius University, SK-83232 Bratislava, Slovakia; lukac@fpharm.uniba.sk
[2] Department of Analytical Chemistry, Faculty of Natural Sciences, Comenius University, SK-84215 Bratislava, Slovakia; josef.jampilek@uniba.sk
[3] Department of Cell and Molecular Biology of Drugs, Faculty of Pharmacy, Comenius University, SK-83232 Bratislava, Slovakia; bilka@fpharm.uniba.sk (F.B.); bilkova@fpharm.uniba.sk (A.B.); paskova@fpharm.uniba.sk (L.P.)
[4] Faculty of Pharmacy, Comenius University, SK-83232 Bratislava, Slovakia; devinsky@fpharm.uniba.sk
[5] Comenius University, SK-81499 Bratislava, Slovakia; renata.horakova@uniba.sk
[6] Materials Research Centre, Faculty of Chemistry, University of Technology, CZ-61200 Brno, Czech Republic; brezina@fch.vut.cz (M.B.); opravil@fch.vutbr.cz (T.O.)
* Correspondence: pisarcik@fpharm.uniba.sk; Tel.: +421-2-50117329

Contents

1. Dynamic light scattering measurements and data analysis
2. Zeta potential measurements
3. SEM high resolution images

1. Dynamic light scattering measurements and data analysis

Screenshots from DLS measurements were obtained using Brookhaven BIC analysis software ver. 6.3 for BI 9000AT digital correlator (DOS version). Five independent measurements were performed for each Ag/surfactant system and each silver-to-surfactant molar ratio value. For each Ag/surfactant system and each molar ratio, the first screenshot provides the image of the autocorrelation function, and the second screenshot provides the data analysis.
The hydrodynamic diameter values of AgNPs shown in the plots in the main article were calculated as the mean of the value Eff.Diam. shown in the second screenshot. This value is calculated from the Taylor series expansion of logarithm of autocorrelation function $g^{(1)}(t)$ in terms of cumulants as follows:

$$\ln(g^{(1)}(t)) = -\bar{\Gamma} t + \frac{1}{2!} \mu_2 t^2 + ...$$

μ_2 is the second cumulant which would be near zero for a perfect single exponential decay. The decay rate $\bar{\Gamma}$ was calculated from the expansion of the logarithm of time correlation function up to the second term using the first linear term in the series for the $\bar{\Gamma}$ calculation (Gam.Ave. in the screenshots). The diffusion coefficient D was determined from the decay rate ($\bar{\Gamma} = D q^2$) and the hydrodynamic diameter of AgNPs (Eff.Diam. in the screenshots) was calculated from the diffusion coefficient using the Stokes–Einstein formula

$$\text{Eff.Diam} = kT / (3\pi\eta D)$$

η is solvent viscosity, k is the Boltzmann constant, and T is absolute temperature. Five independent measurements and calculations of time correlation function were carried out for each Ag/surfactant system and silver-to-surfactant molar ratio investigated. The mean value and standard deviation of Eff.Diam for each sample were calculated and plotted in the graphs.

To reduce the excessive number of screenshots in this supplementary material, just one independent measurement for each Ag/surfactant system and each silver-to-surfactant molar ratio value is shown.

Screenshots section - DLS

Ag/14pl, n_{Ag}/n_{Surf} = 8

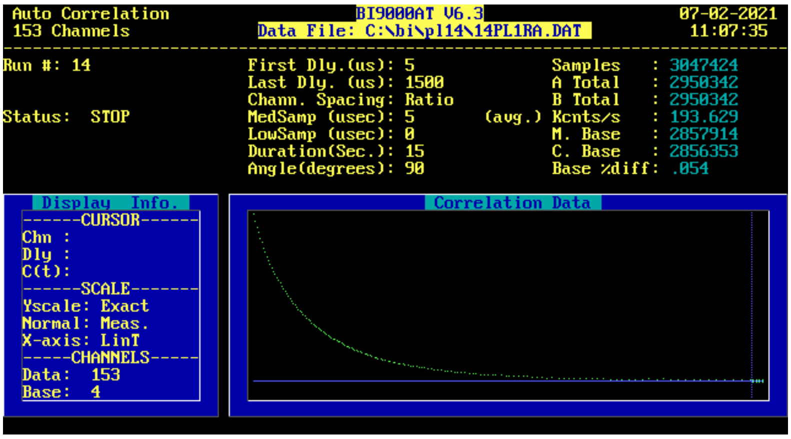

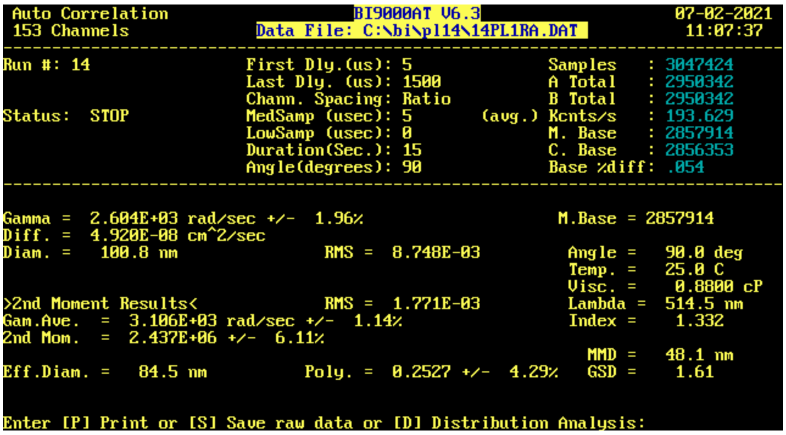

Ag/14pl, n_{Ag}/n_{Surf} = 4

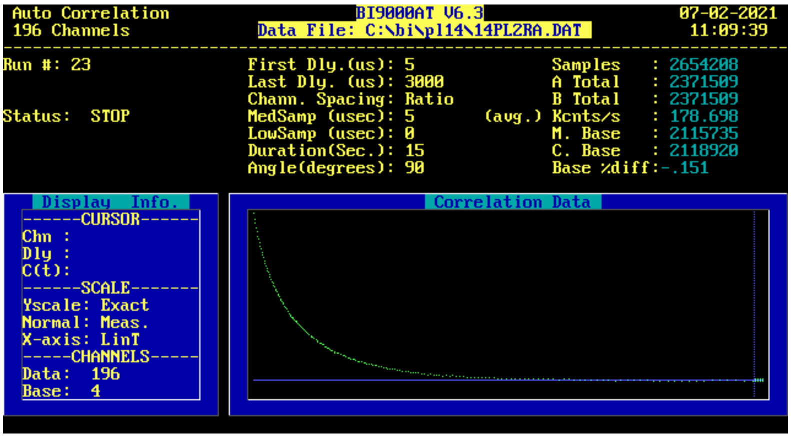

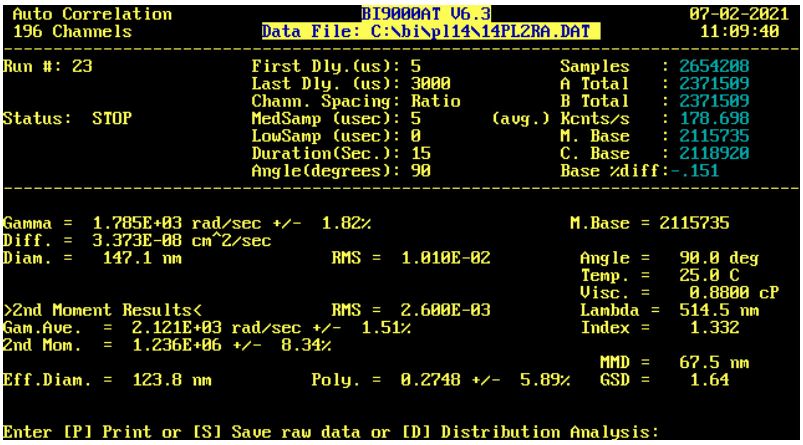

Ag/14pl, n_{Ag}/n_{Surf} = 2.7

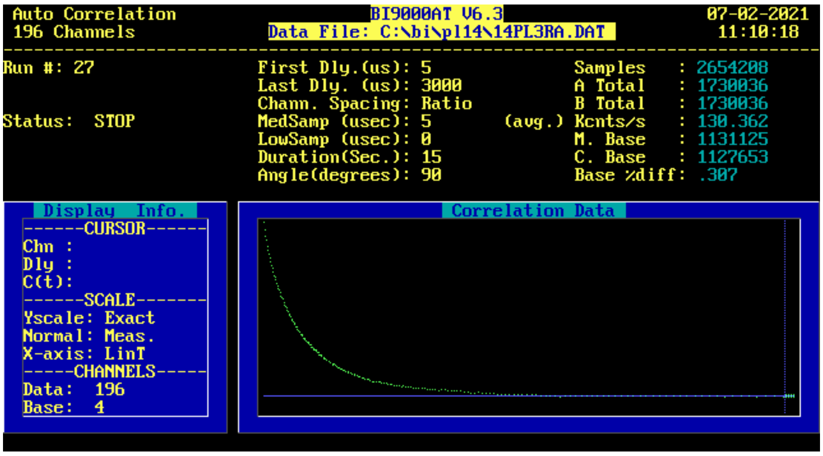

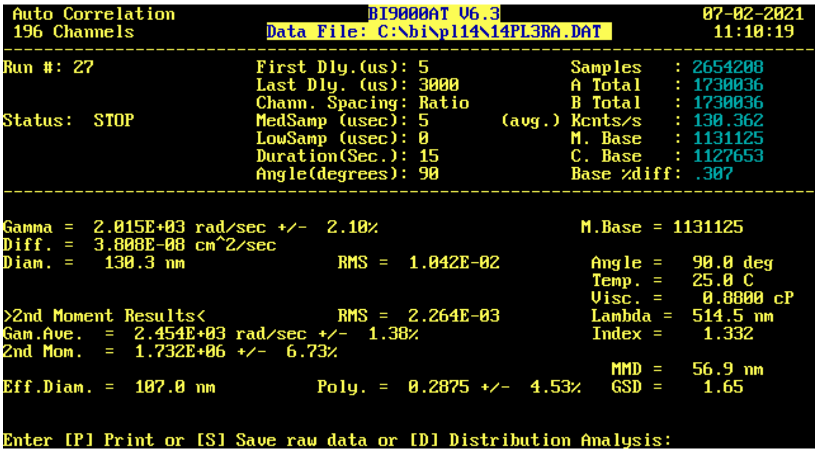

Ag/14pl, n_{Ag}/n_{Surf} = 2

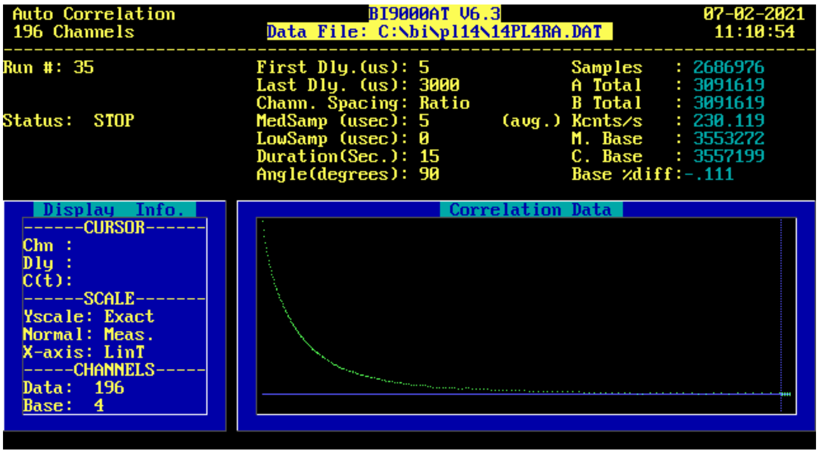

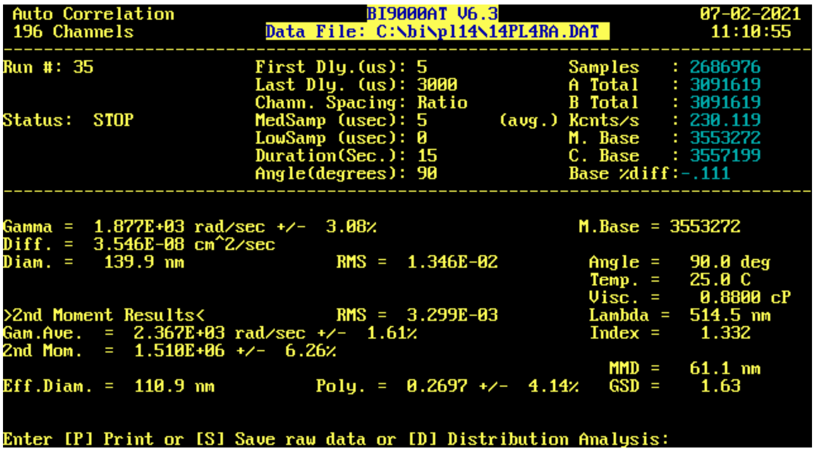

Ag/15pl, n_{Ag}/n_{Surf} = 8

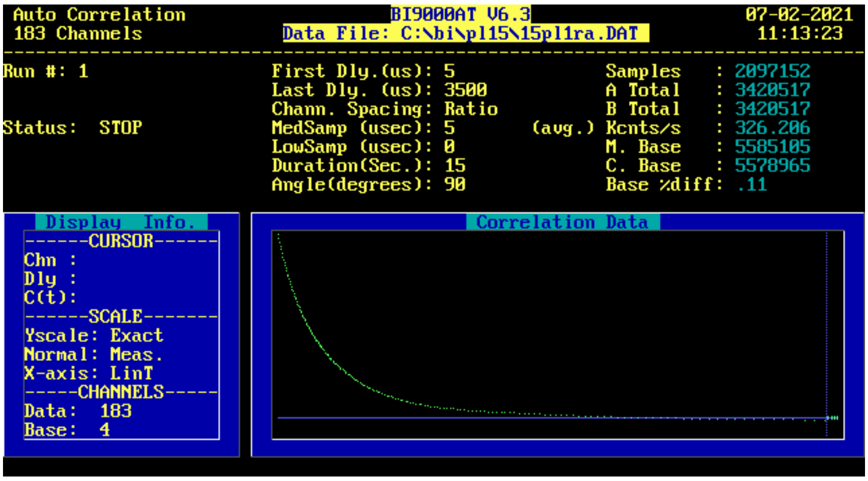

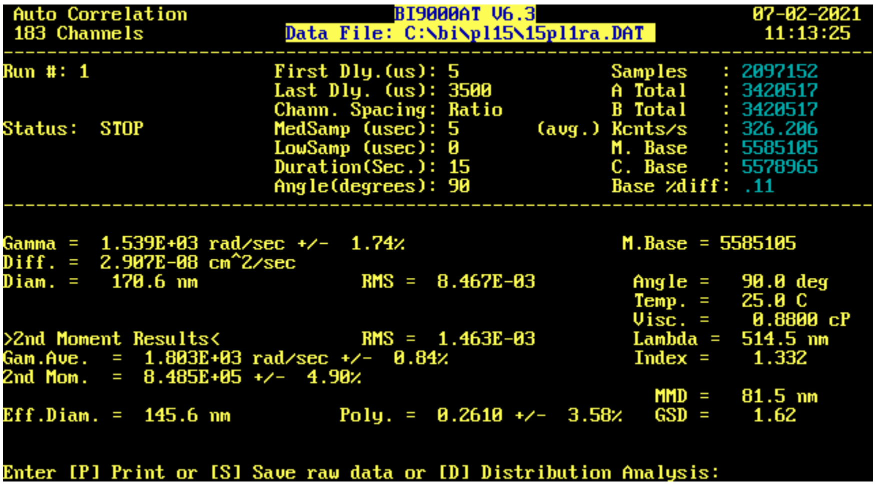

Ag/15pl, n_{Ag}/n_{Surf} = 4

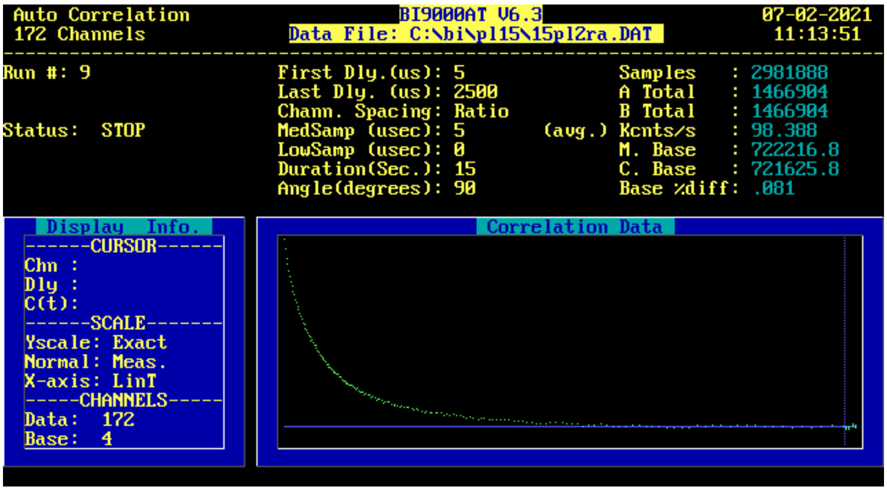

```
 Auto Correlation                    BI9000AT V6.3                            07-02-2021
 172 Channels                  Data File: C:\bi\pl15\15pl2ra.DAT               11:13:52
--------------------------------------------------------------------------------
Run #: 9                  First Dly.(us): 5               Samples    : 2981888
                          Last Dly. (us): 2500            A Total    : 1466904
                          Chann. Spacing: Ratio           B Total    : 1466904
Status:  STOP             MedSamp (usec): 5        (avg.) Kcnts/s    : 98.388
                          LowSamp (usec): 0               M. Base    : 722216.8
                          Duration(Sec.): 15              C. Base    : 721625.8
                          Angle(degrees): 90              Base %diff: .081
--------------------------------------------------------------------------------

Gamma =  2.188E+03 rad/sec +/-  3.32%                    M.Base = 722216.8
Diff. =  4.134E-08 cm^2/sec
Diam. =   120.0 nm                RMS =  9.243E-03         Angle =   90.0 deg
                                                           Temp. =   25.0 C
                                                           Visc. =    0.8800 cP
>2nd Moment Results<              RMS =  2.236E-03         Lambda =  514.5 nm
Gam.Ave.  =  2.885E+03 rad/sec +/-  2.19%                  Index =    1.332
2nd Mom.  =  2.761E+06 +/-  7.88%
                                                             MMD =   44.5 nm
Eff.Diam. =   91.0 nm          Poly. = 0.3318 +/-  4.61%   GSD =    1.71


Enter [P] Print or [S] Save raw data or [D] Distribution Analysis:
```

Ag/15pl, n_{Ag}/n_{Surf} = 2.7

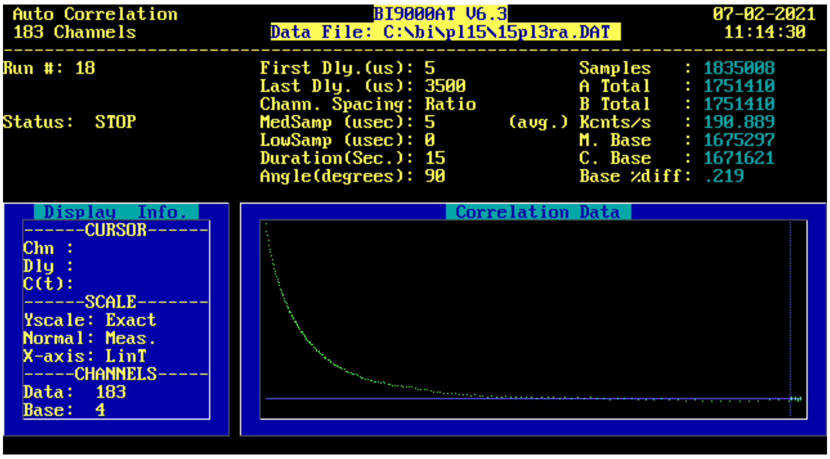

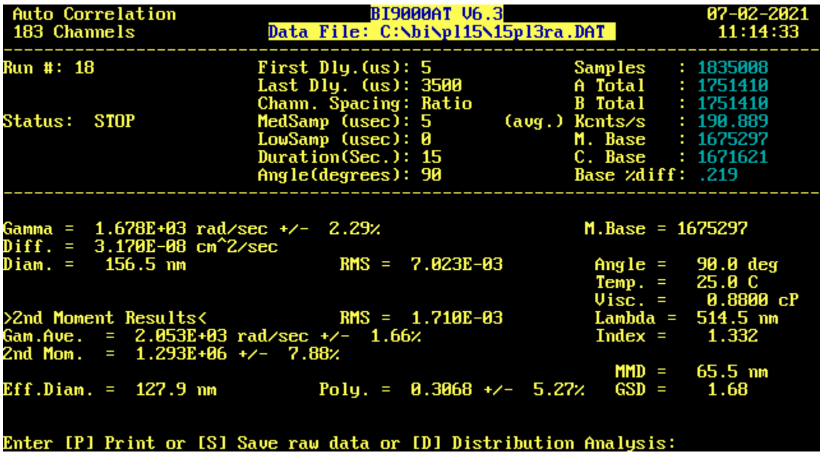

```
Auto Correlation                BI9000AT V6.3                 07-02-2021
183 Channels           Data File: C:\bi\pl15\15pl3ra.DAT        11:14:33
Run #: 18              First Dly.(us): 5              Samples   : 1835008
                       Last Dly. (us): 3500           A Total   : 1751410
                       Chann. Spacing: Ratio          B Total   : 1751410
Status:  STOP          MedSamp (usec): 5       (avg.) Kcnts/s   : 190.889
                       LowSamp (usec): 0              M. Base   : 1675297
                       Duration(Sec.): 15             C. Base   : 1671621
                       Angle(degrees): 90             Base %diff: .219

Gamma =  1.678E+03 rad/sec +/-  2.29%            M.Base = 1675297
Diff. =  3.170E-08 cm^2/sec
Diam. =   156.5 nm          RMS =  7.023E-03      Angle =   90.0 deg
                                                  Temp. =   25.0 C
                                                  Visc. =    0.8800 cP
>2nd Moment Results<        RMS =  1.710E-03      Lambda =  514.5 nm
Gam.Ave.  =  2.053E+03 rad/sec +/-  1.66%         Index =    1.332
2nd Mom.  =  1.293E+06 +/-  7.88%
                                                    MMD =   65.5 nm
Eff.Diam. =  127.9 nm        Poly. = 0.3068 +/-  5.27%  GSD =  1.68

Enter [P] Print or [S] Save raw data or [D] Distribution Analysis:
```

Ag/15pl, n_{Ag}/n_{Surf} = 2

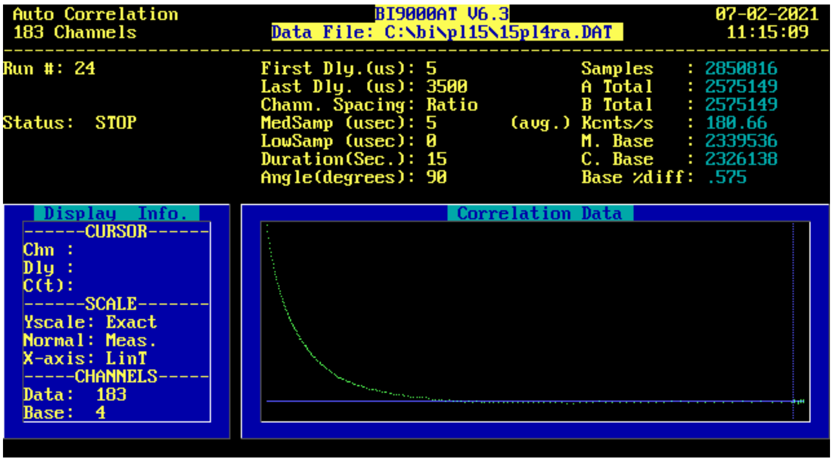

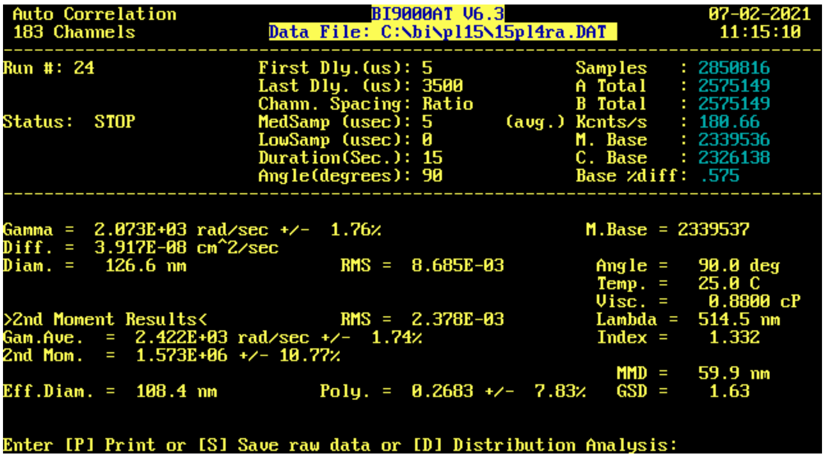

```
Auto Correlation                BI9000AT V6.3                 07-02-2021
183 Channels           Data File: C:\bi\pl15\15pl4ra.DAT        11:15:10
Run #: 24              First Dly.(us): 5              Samples   : 2850816
                       Last Dly. (us): 3500           A Total   : 2575149
                       Chann. Spacing: Ratio          B Total   : 2575149
Status:  STOP          MedSamp (usec): 5       (avg.) Kcnts/s   : 180.66
                       LowSamp (usec): 0              M. Base   : 2339536
                       Duration(Sec.): 15             C. Base   : 2326138
                       Angle(degrees): 90             Base %diff: .575

Gamma =  2.073E+03 rad/sec +/-  1.76%            M.Base = 2339537
Diff. =  3.917E-08 cm^2/sec
Diam. =   126.6 nm          RMS =  8.685E-03      Angle =   90.0 deg
                                                  Temp. =   25.0 C
                                                  Visc. =    0.8800 cP
>2nd Moment Results<        RMS =  2.378E-03      Lambda =  514.5 nm
Gam.Ave.  =  2.422E+03 rad/sec +/-  1.74%         Index =    1.332
2nd Mom.  =  1.573E+06 +/- 10.77%
                                                    MMD =   59.9 nm
Eff.Diam. =  108.4 nm        Poly. = 0.2683 +/-  7.83%  GSD =  1.63

Enter [P] Print or [S] Save raw data or [D] Distribution Analysis:
```

Ag/16pl, n_{Ag}/n_{Surf} = 8

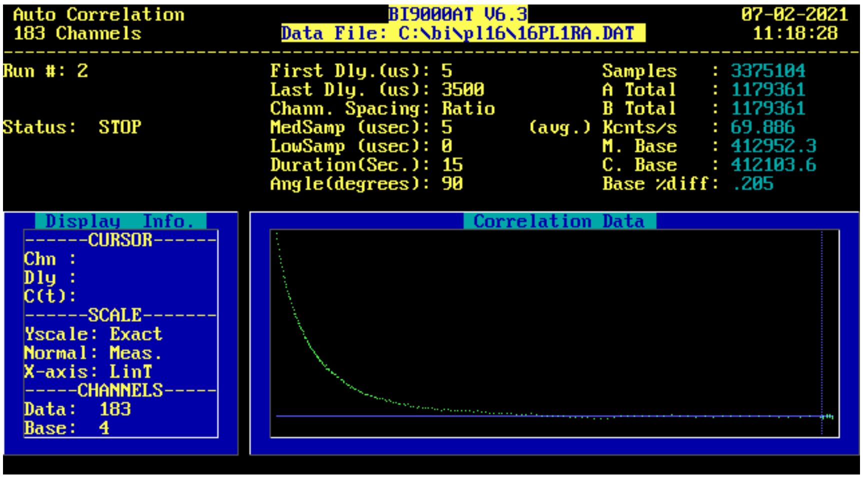

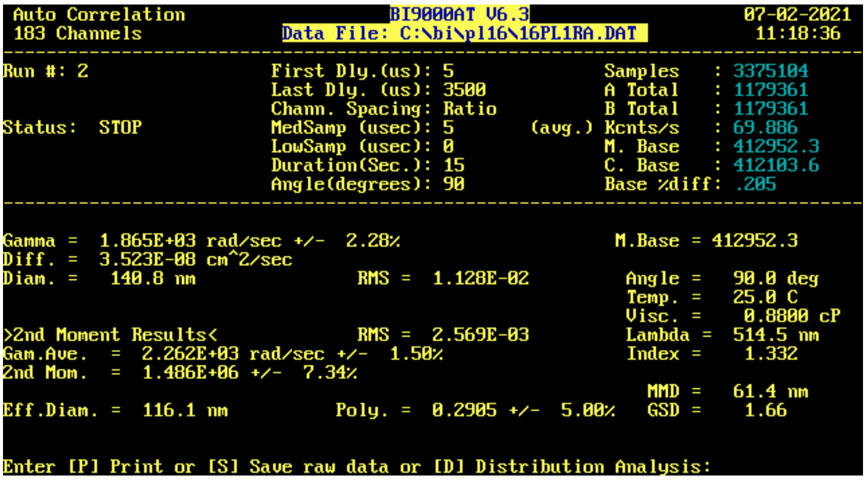

Ag/16pl, n_{Ag}/n_{Surf} = 4

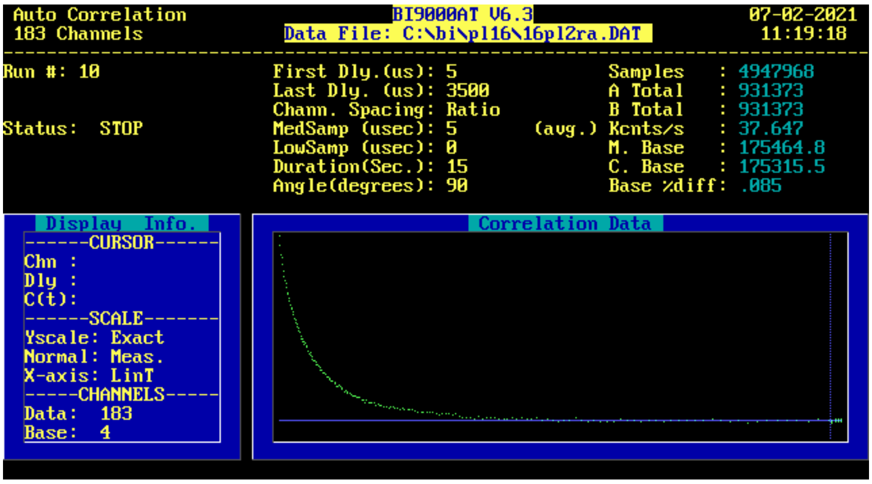

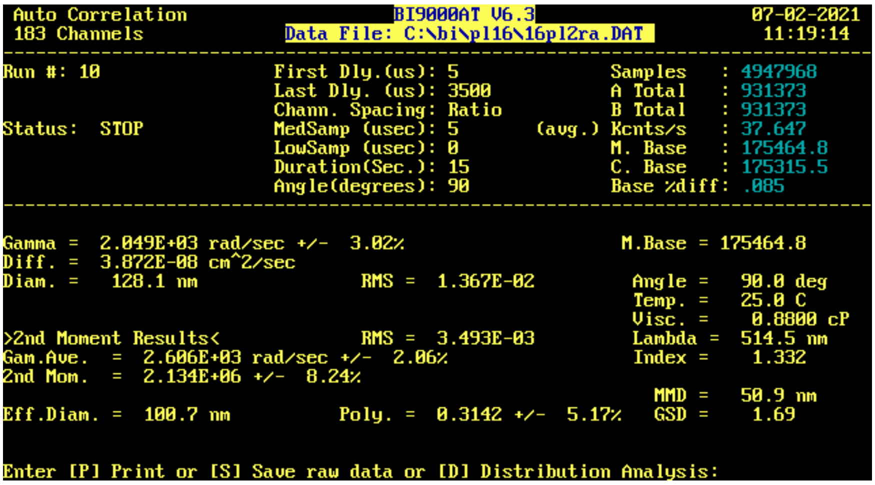

```
 Auto Correlation                   BI9000AT V6.3                    07-02-2021
 183 Channels             Data File: C:\bi\pl16\16pl2ra.DAT             11:19:14
--------------------------------------------------------------------------------
Run #: 10                 First Dly.(us): 5              Samples    : 4947968
                          Last Dly. (us): 3500           A Total    : 931373
                          Chann. Spacing: Ratio          B Total    : 931373
Status:  STOP             MedSamp (usec): 5        (avg.) Kcnts/s   : 37.647
                          LowSamp (usec): 0              M. Base    : 175464.8
                          Duration(Sec.): 15             C. Base    : 175315.5
                          Angle(degrees): 90             Base %diff: .085
--------------------------------------------------------------------------------

Gamma =  2.049E+03 rad/sec +/-  3.02%                      M.Base = 175464.8
Diff. =  3.872E-08 cm^2/sec
Diam. =   128.1 nm                RMS =  1.367E-02          Angle =   90.0 deg
                                                            Temp. =   25.0 C
                                                            Visc. =    0.8800 cP
>2nd Moment Results<              RMS =  3.493E-03          Lambda =  514.5 nm
Gam.Ave.  =  2.606E+03 rad/sec +/-  2.06%                   Index =    1.332
2nd Mom.  =  2.134E+06 +/-  8.24%
                                                              MMD =   50.9 nm
Eff.Diam. =  100.7 nm          Poly. = 0.3142 +/-  5.17%    GSD =    1.69

Enter [P] Print or [S] Save raw data or [D] Distribution Analysis:
```

Ag/16pl, n_{Ag}/n_{Surf} = 2.7

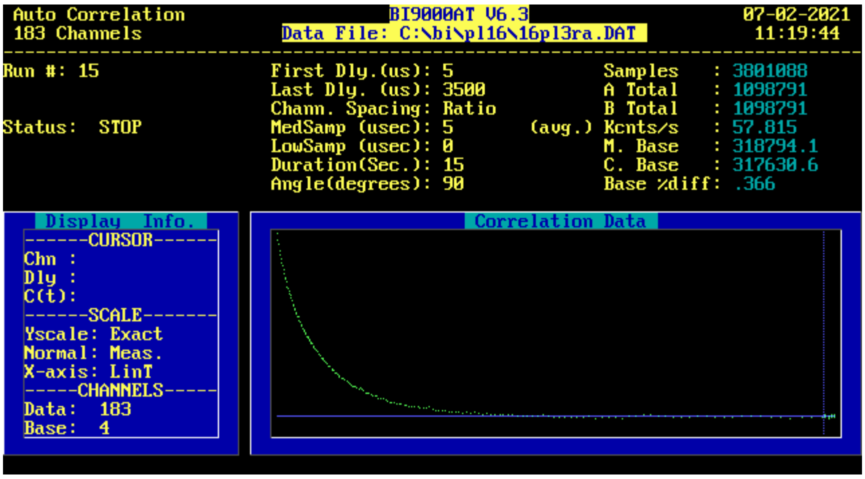

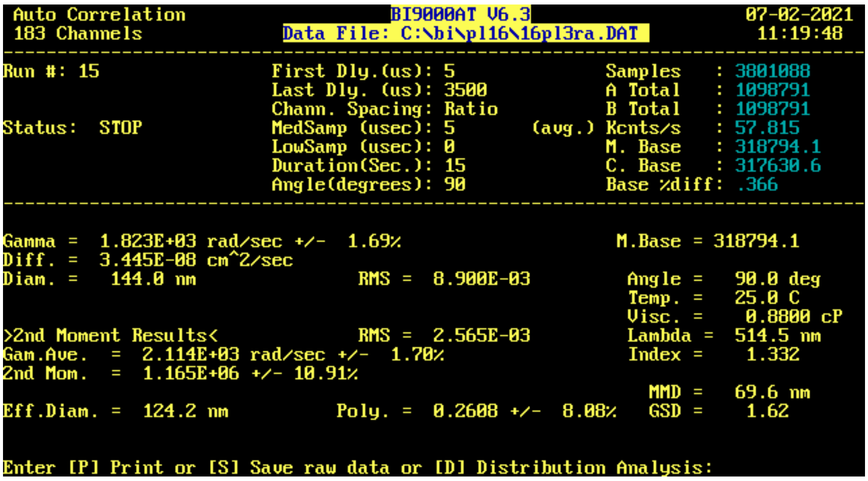

Ag/16pl, n_{Ag}/n_{Surf} = 2

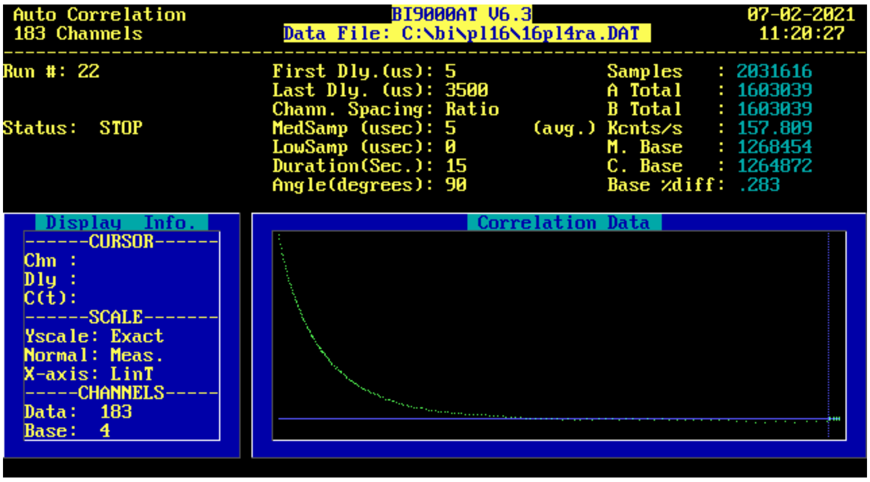

```
Auto Correlation                       BI9000AT V6.3                       07-02-2021
183 Channels                  Data File: C:\bi\pl16\16pl4ra.DAT             11:20:28
-------------------------------------------------------------------------------------
Run #: 22                 First Dly.(us): 5              Samples     : 2031616
                          Last Dly. (us): 3500           A Total     : 1603039
                          Chann. Spacing: Ratio          B Total     : 1603039
Status:  STOP             MedSamp (usec): 5        (avg.) Kcnts/s    : 157.809
                          LowSamp (usec): 0              M. Base     : 1268454
                          Duration(Sec.): 15             C. Base     : 1264872
                          Angle(degrees): 90             Base %diff: .283
-------------------------------------------------------------------------------------

Gamma =  1.677E+03 rad/sec +/-  1.25%                        M.Base = 1268454
Diff. =  3.169E-08 cm^2/sec
Diam. =   156.6 nm                RMS =  7.006E-03           Angle =    90.0 deg
                                                             Temp. =    25.0 C
                                                             Visc. =     0.8800 cP
>2nd Moment Results<              RMS =  1.790E-03           Lambda =  514.5 nm
Gam.Ave.  =  1.884E+03 rad/sec +/-  1.17%                    Index =     1.332
2nd Mom.  =  7.922E+05 +/-  9.45%
                                                               MMD =    84.2 nm
Eff.Diam. =  139.4 nm          Poly. =  0.2232 +/-  7.46%      GSD =     1.57

Enter [P] Print or [S] Save raw data or [D] Distribution Analysis:
```

Ag/17pl, n_{Ag}/n_{Surf} = 8

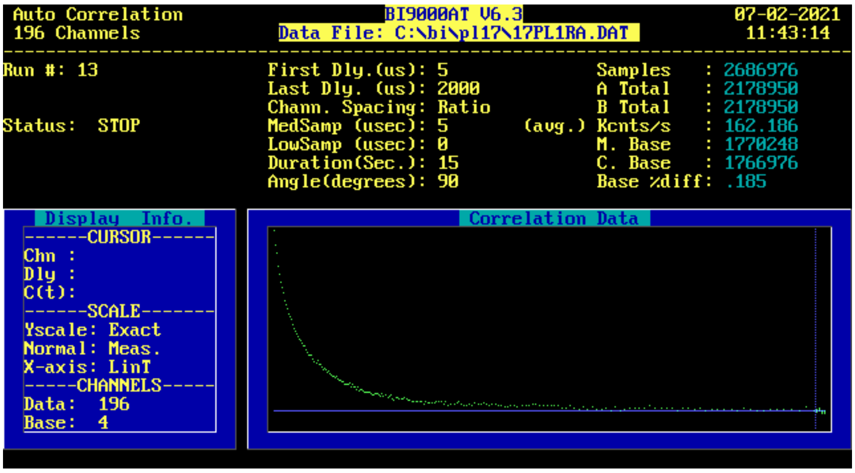
Auto Correlation BI9000AT V6.3 07-02-2021
196 Channels Data File: C:\bi\pl17\17PL1RA.DAT 11:43:14

Run #: 13 First Dly.(us): 5 Samples : 2686976
Last Dly. (us): 2000 A Total : 2178950
Chann. Spacing: Ratio B Total : 2178950
Status: STOP MedSamp (usec): 5 (avg.) Kcnts/s : 162.186
LowSamp (usec): 0 M. Base : 1770248
Duration(Sec.): 15 C. Base : 1766976
Angle(degrees): 90 Base %diff: .185

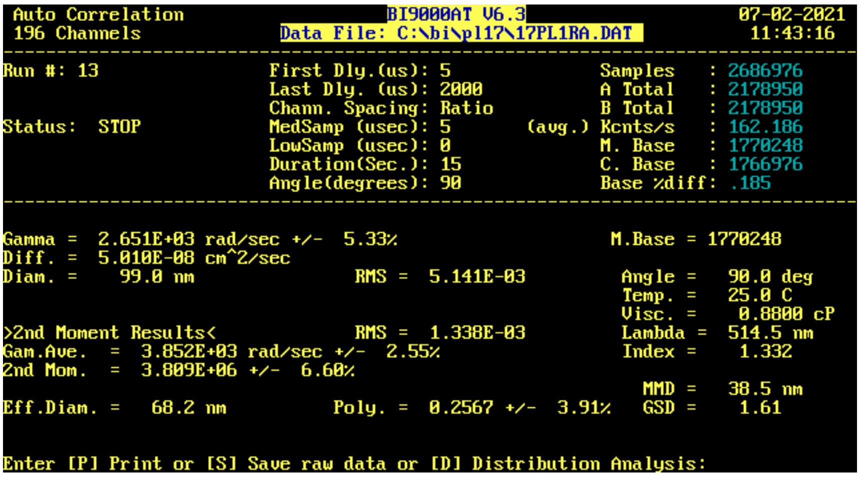
Auto Correlation BI9000AT V6.3 07-02-2021
196 Channels Data File: C:\bi\pl17\17PL1RA.DAT 11:43:16

Run #: 13 First Dly.(us): 5 Samples : 2686976
Last Dly. (us): 2000 A Total : 2178950
Chann. Spacing: Ratio B Total : 2178950
Status: STOP MedSamp (usec): 5 (avg.) Kcnts/s : 162.186
LowSamp (usec): 0 M. Base : 1770248
Duration(Sec.): 15 C. Base : 1766976
Angle(degrees): 90 Base %diff: .185

Gamma = 2.651E+03 rad/sec +/- 5.33% M.Base = 1770248
Diff. = 5.010E-08 cm^2/sec
Diam. = 99.0 nm RMS = 5.141E-03 Angle = 90.0 deg
Temp. = 25.0 C
Visc. = 0.8800 cP
>2nd Moment Results< RMS = 1.338E-03 Lambda = 514.5 nm
Gam.Ave. = 3.852E+03 rad/sec +/- 2.55% Index = 1.332
2nd Mom. = 3.809E+06 +/- 6.60%
MMD = 38.5 nm
Eff.Diam. = 68.2 nm Poly. = 0.2567 +/- 3.91% GSD = 1.61

Enter [P] Print or [S] Save raw data or [D] Distribution Analysis:

Ag/17pl, n_{Ag}/n_{Surf} = 4

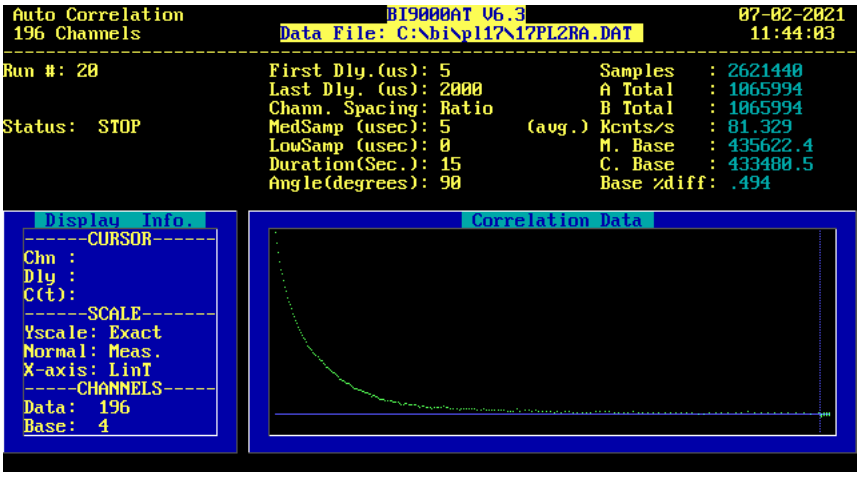
Auto Correlation BI9000AT V6.3 07-02-2021
196 Channels Data File: C:\bi\pl17\17PL2RA.DAT 11:44:03

Run #: 20 First Dly.(us): 5 Samples : 2621440
Last Dly. (us): 2000 A Total : 1065994
Chann. Spacing: Ratio B Total : 1065994
Status: STOP MedSamp (usec): 5 (avg.) Kcnts/s : 81.329
LowSamp (usec): 0 M. Base : 435622.4
Duration(Sec.): 15 C. Base : 433480.5
Angle(degrees): 90 Base %diff: .494

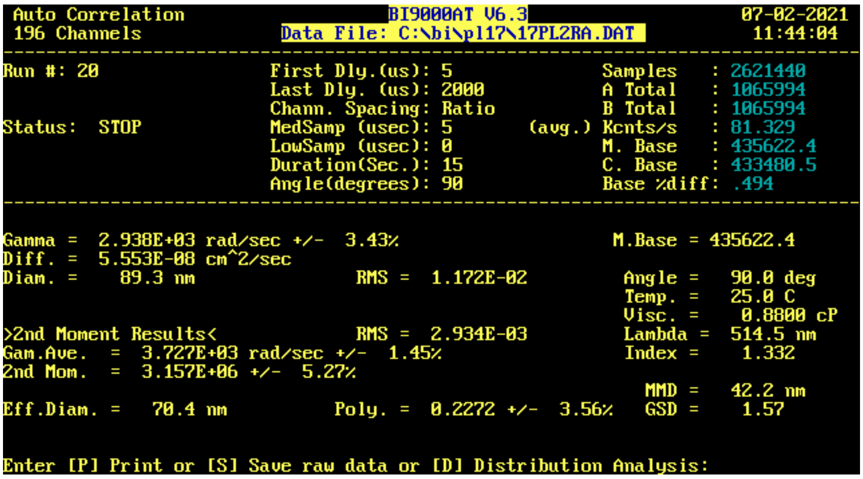
Auto Correlation BI9000AT V6.3 07-02-2021
196 Channels Data File: C:\bi\pl17\17PL2RA.DAT 11:44:04

Run #: 20 First Dly.(us): 5 Samples : 2621440
Last Dly. (us): 2000 A Total : 1065994
Chann. Spacing: Ratio B Total : 1065994
Status: STOP MedSamp (usec): 5 (avg.) Kcnts/s : 81.329
LowSamp (usec): 0 M. Base : 435622.4
Duration(Sec.): 15 C. Base : 433480.5
Angle(degrees): 90 Base %diff: .494

Gamma = 2.938E+03 rad/sec +/- 3.43% M.Base = 435622.4
Diff. = 5.553E-08 cm^2/sec
Diam. = 89.3 nm RMS = 1.172E-02 Angle = 90.0 deg
Temp. = 25.0 C
Visc. = 0.8800 cP
>2nd Moment Results< RMS = 2.934E-03 Lambda = 514.5 nm
Gam.Ave. = 3.727E+03 rad/sec +/- 1.45% Index = 1.332
2nd Mom. = 3.157E+06 +/- 5.27%
MMD = 42.2 nm
Eff.Diam. = 70.4 nm Poly. = 0.2272 +/- 3.56% GSD = 1.57

Enter [P] Print or [S] Save raw data or [D] Distribution Analysis:

Ag/17pl, n_{Ag}/n_{Surf} = 2.7

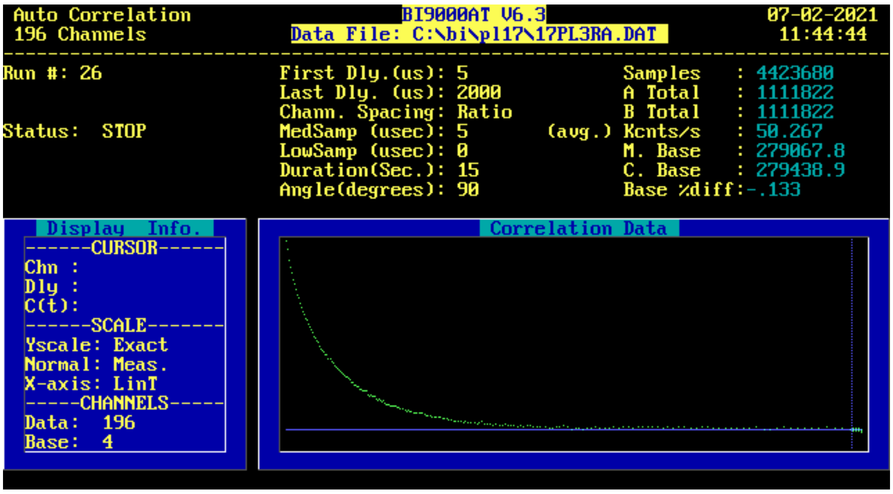

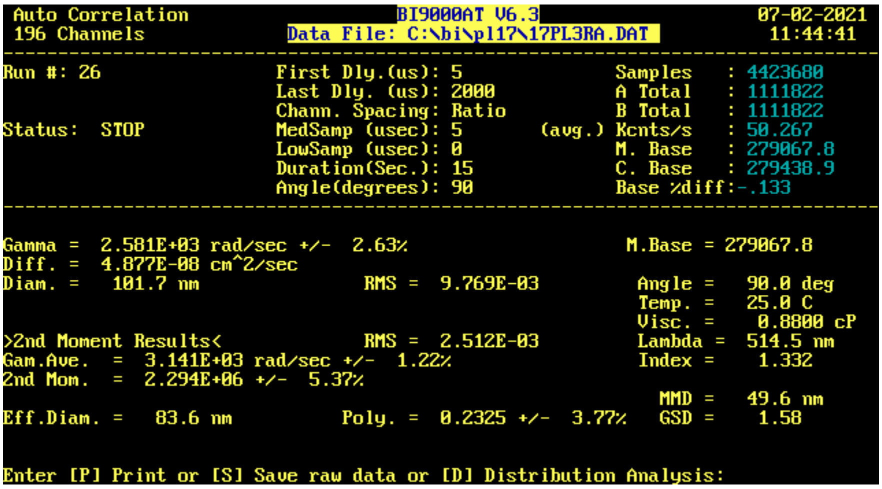

Ag/17pl, n_{Ag}/n_{Surf} = 2

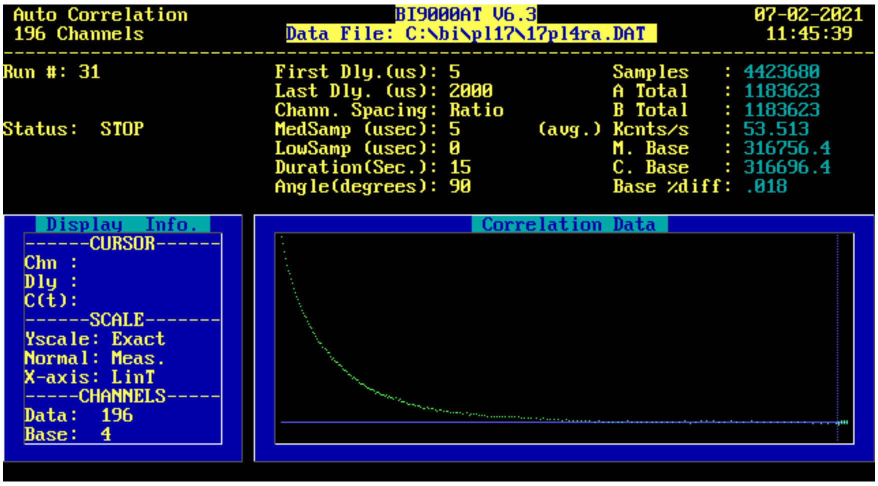

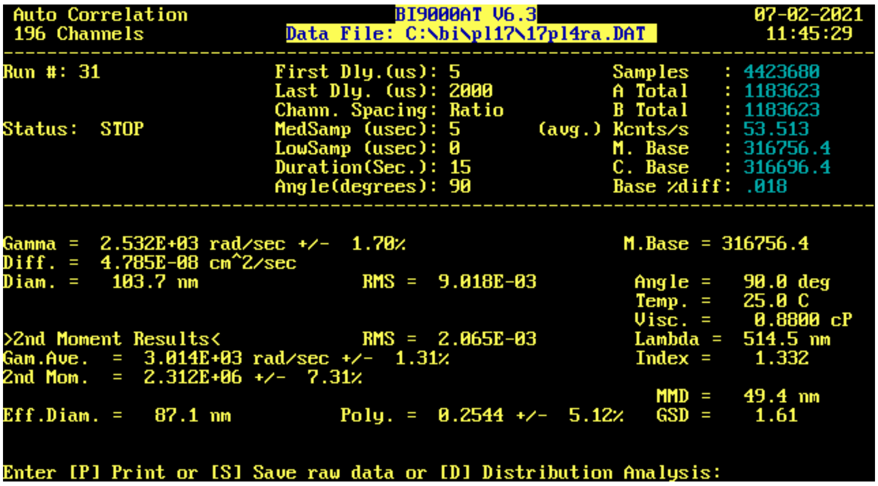
Auto Correlation
196 Channels
BI9000AT V6.3
Data File: C:\bi\pl17\17pl4ra.DAT
07-02-2021
11:45:29
Run #: 31
Status: STOP
First Dly.(us): 5
Last Dly. (us): 2000
Chann. Spacing: Ratio
MedSamp (usec): 5
LowSamp (usec): 0
Duration(Sec.): 15
Angle(degrees): 90
Samples : 4423680
A Total : 1183623
B Total : 1183623
(avg.) Kcnts/s : 53.513
M. Base : 316756.4
C. Base : 316696.4
Base %diff: .018
Gamma = 2.532E+03 rad/sec +/- 1.70%
Diff. = 4.785E-08 cm^2/sec
Diam. = 103.7 nm
RMS = 9.018E-03
M.Base = 316756.4
Angle = 90.0 deg
Temp. = 25.0 C
Visc. = 0.8800 cP
Lambda = 514.5 nm
Index = 1.332
>2nd Moment Results<
RMS = 2.065E-03
Gam.Ave. = 3.014E+03 rad/sec +/- 1.31%
2nd Mom. = 2.312E+06 +/- 7.31%
MMD = 49.4 nm
Eff.Diam. = 87.1 nm
Poly. = 0.2544 +/- 5.12%
GSD = 1.61
Enter [P] Print or [S] Save raw data or [D] Distribution Analysis:

Ag/18pl, n_{Ag}/n_{Surf} = 8

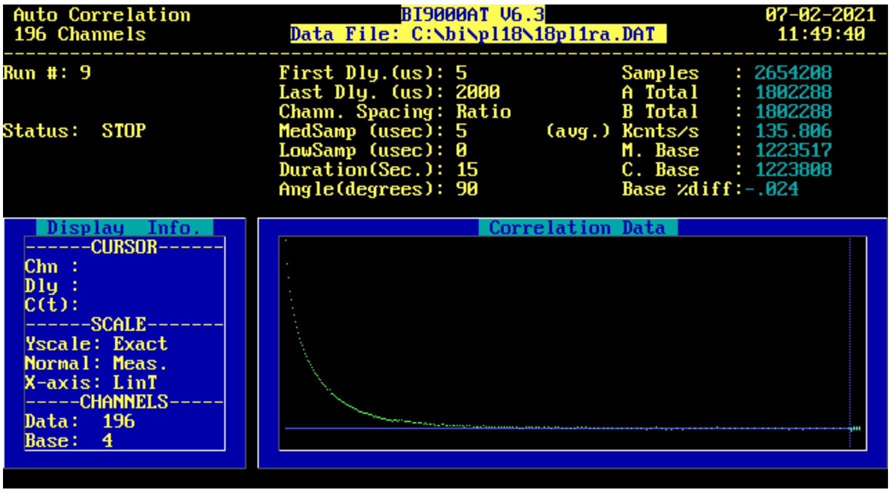

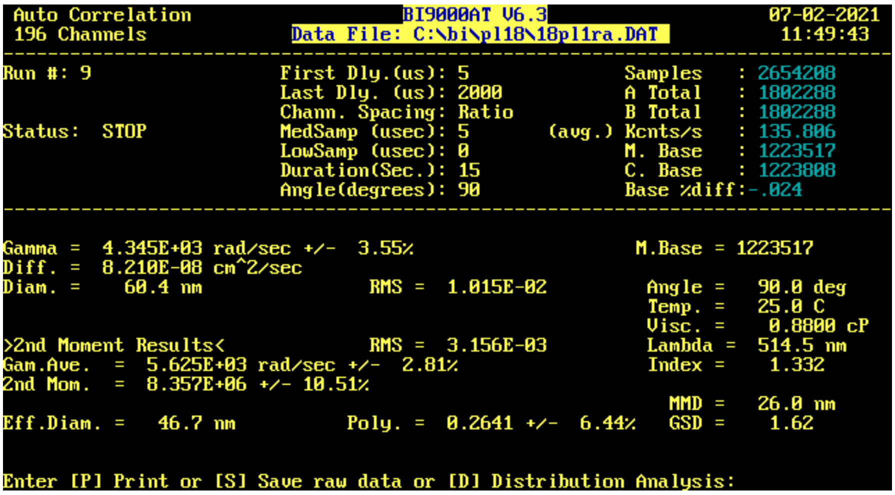

Ag/18pl, n_{Ag}/n_{Surf} = 4

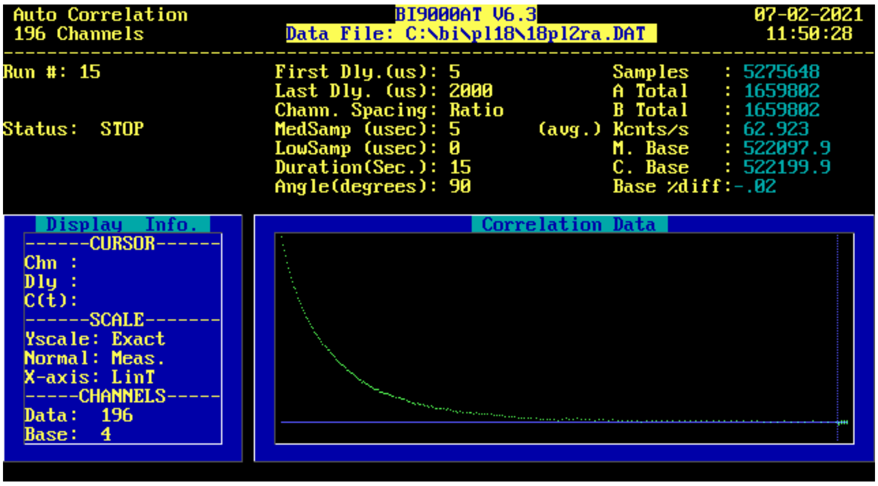

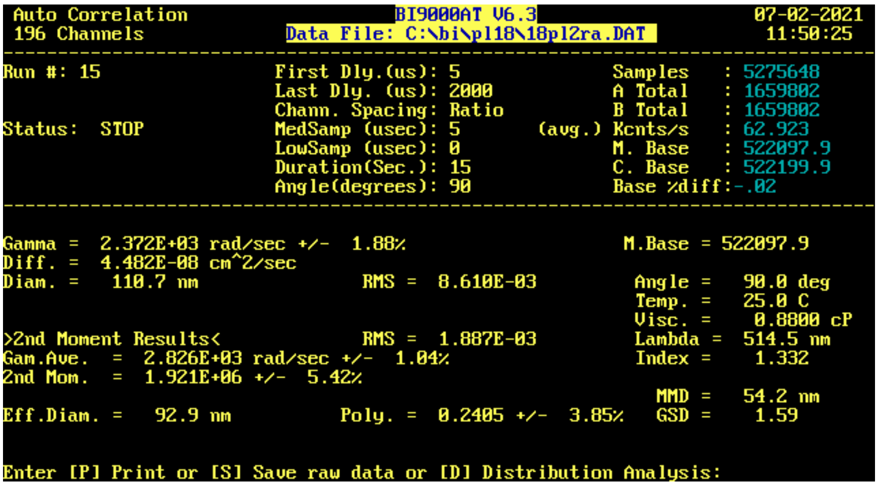

```
 Auto Correlation                     BI9000AT V6.3                           07-02-2021
 196 Channels               Data File: C:\bi\pl18\18pl2ra.DAT                   11:50:25
--------------------------------------------------------------------------------
Run #: 15                 First Dly.(us): 5            Samples    : 5275648
                          Last Dly. (us): 2000         A Total    : 1659802
                          Chann. Spacing: Ratio        B Total    : 1659802
Status:  STOP             MedSamp (usec): 5      (avg.) Kcnts/s   : 62.923
                          LowSamp (usec): 0            M. Base    : 522097.9
                          Duration(Sec.): 15           C. Base    : 522199.9
                          Angle(degrees): 90           Base %diff:-.02
--------------------------------------------------------------------------------

Gamma =  2.372E+03 rad/sec +/-  1.88%                   M.Base = 522097.9
Diff. =  4.482E-08 cm^2/sec
Diam. =   110.7 nm                RMS =  8.610E-03         Angle =   90.0 deg
                                                           Temp. =   25.0 C
                                                           Visc. =    0.8800 cP
>2nd Moment Results<              RMS =  1.887E-03         Lambda =  514.5 nm
Gam.Ave.  =  2.826E+03 rad/sec +/-  1.04%                  Index =    1.332
2nd Mom.  =  1.921E+06 +/-  5.42%
                                                             MMD =   54.2 nm
Eff.Diam. =   92.9 nm           Poly. = 0.2405 +/-  3.85%   GSD =    1.59

Enter [P] Print or [S] Save raw data or [D] Distribution Analysis:
```

Ag/18pl, n_{Ag}/n_{Surf} = 2.7

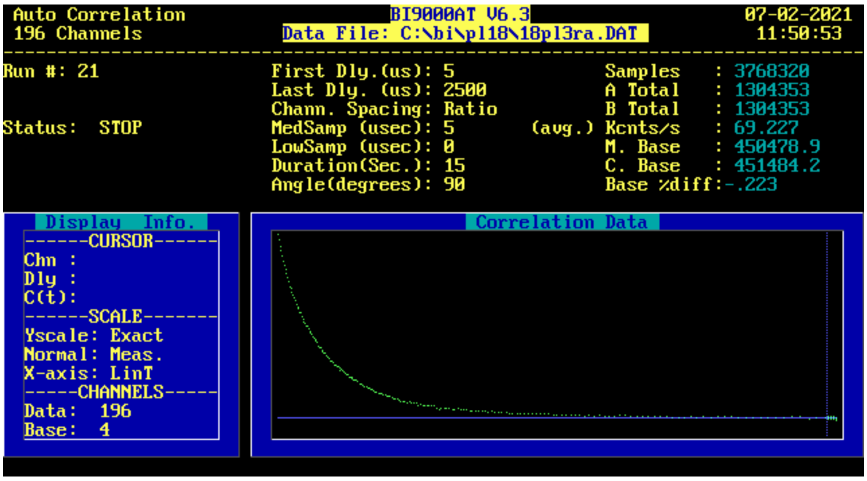
Auto Correlation BI9000AT V6.3 07-02-2021
196 Channels Data File: C:\bi\pl18\18pl3ra.DAT 11:50:53

Run #: 21 First Dly.(us): 5 Samples : 3768320
Last Dly. (us): 2500 A Total : 1304353
Chann. Spacing: Ratio B Total : 1304353
Status: STOP MedSamp (usec): 5 (avg.) Kcnts/s : 69.227
LowSamp (usec): 0 M. Base : 450478.9
Duration(Sec.): 15 C. Base : 451484.2
Angle(degrees): 90 Base %diff:-.223

Display Info.
------CURSOR------
Chn :
Dly :
C(t):
------SCALE-------
Yscale: Exact
Normal: Meas.
X-axis: LinT
-----CHANNELS-----
Data: 196
Base: 4

Correlation Data

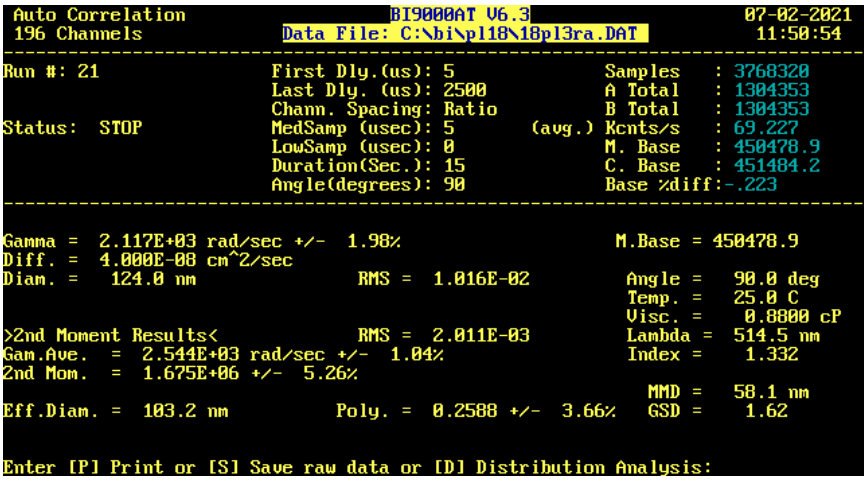
Auto Correlation BI9000AT V6.3 07-02-2021
196 Channels Data File: C:\bi\pl18\18pl3ra.DAT 11:50:54

Run #: 21 First Dly.(us): 5 Samples : 3768320
Last Dly. (us): 2500 A Total : 1304353
Chann. Spacing: Ratio B Total : 1304353
Status: STOP MedSamp (usec): 5 (avg.) Kcnts/s : 69.227
LowSamp (usec): 0 M. Base : 450478.9
Duration(Sec.): 15 C. Base : 451484.2
Angle(degrees): 90 Base %diff:-.223

Gamma = 2.117E+03 rad/sec +/- 1.98% M.Base = 450478.9
Diff. = 4.000E-08 cm^2/sec
Diam. = 124.0 nm RMS = 1.016E-02 Angle = 90.0 deg
Temp. = 25.0 C
Visc. = 0.8800 cP
>2nd Moment Results< RMS = 2.011E-03 Lambda = 514.5 nm
Gam.Ave. = 2.544E+03 rad/sec +/- 1.04% Index = 1.332
2nd Mom. = 1.675E+06 +/- 5.26%
MMD = 58.1 nm
Eff.Diam. = 103.2 nm Poly. = 0.2588 +/- 3.66% GSD = 1.62

Enter [P] Print or [S] Save raw data or [D] Distribution Analysis:

Ag/18pl, n_{Ag}/n_{Surf} = 2

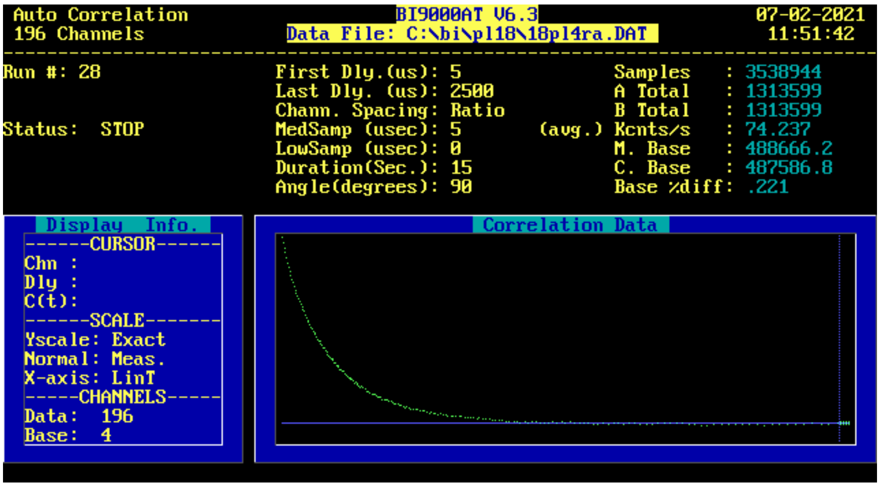
Auto Correlation BI9000AT V6.3 07-02-2021
196 Channels Data File: C:\bi\pl18\18pl4ra.DAT 11:51:42

Run #: 28 First Dly.(us): 5 Samples : 3538944
Last Dly. (us): 2500 A Total : 1313599
Chann. Spacing: Ratio B Total : 1313599
Status: STOP MedSamp (usec): 5 (avg.) Kcnts/s : 74.237
LowSamp (usec): 0 M. Base : 488666.2
Duration(Sec.): 15 C. Base : 487586.8
Angle(degrees): 90 Base %diff: .221

Display Info.
------CURSOR------
Chn :
Dly :
C(t):
------SCALE-------
Yscale: Exact
Normal: Meas.
X-axis: LinT
-----CHANNELS-----
Data: 196
Base: 4

Correlation Data

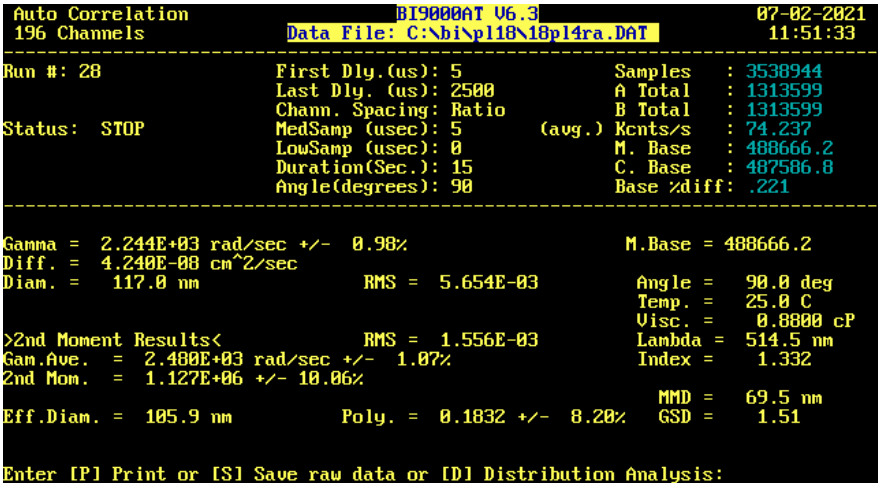
Auto Correlation BI9000AT V6.3 07-02-2021
196 Channels Data File: C:\bi\pl18\18pl4ra.DAT 11:51:33

Run #: 28 First Dly.(us): 5 Samples : 3538944
Last Dly. (us): 2500 A Total : 1313599
Chann. Spacing: Ratio B Total : 1313599
Status: STOP MedSamp (usec): 5 (avg.) Kcnts/s : 74.237
LowSamp (usec): 0 M. Base : 488666.2
Duration(Sec.): 15 C. Base : 487586.8
Angle(degrees): 90 Base %diff: .221

Gamma = 2.244E+03 rad/sec +/- 0.98% M.Base = 488666.2
Diff. = 4.240E-08 cm^2/sec
Diam. = 117.0 nm RMS = 5.654E-03 Angle = 90.0 deg
Temp. = 25.0 C
Visc. = 0.8800 cP
>2nd Moment Results< RMS = 1.556E-03 Lambda = 514.5 nm
Gam.Ave. = 2.480E+03 rad/sec +/- 1.07% Index = 1.332
2nd Mom. = 1.127E+06 +/- 10.06%
MMD = 69.5 nm
Eff.Diam. = 105.9 nm Poly. = 0.1832 +/- 8.20% GSD = 1.51

Enter [P] Print or [S] Save raw data or [D] Distribution Analysis:

Image of the used DLS experimental setup:

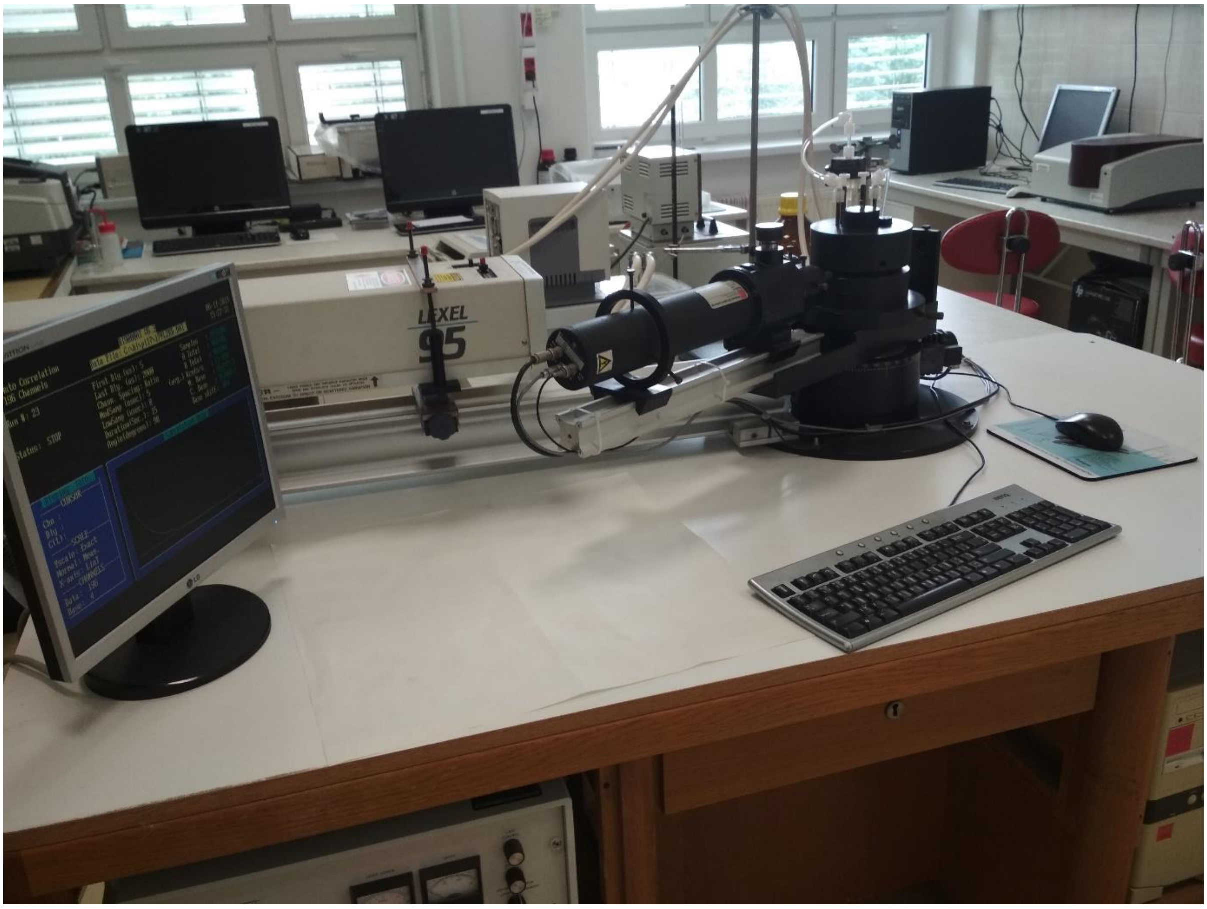

2. Zeta potential measurements

Screenshots of zeta potential peaks and the set of 10 repeated measurements are shown for each Ag/surfactant system and each silver-to-surfactant molar ratio value. The screenshots were obtained from the Broohkaven Instruments Zeta Potential Analyzer Software ver. 5.68. To reduce the excessive number of screenshots in the supplementary material, just single set of 10 independent readings of zeta potential for each Ag/surfactant system and each silver-to-surfactant molar ratio value is shown.

Screenshots section – zeta potential

Ag/14pl

Ag/14pl nAg/nSurf = 8 (Run 10)

Measurement Completed

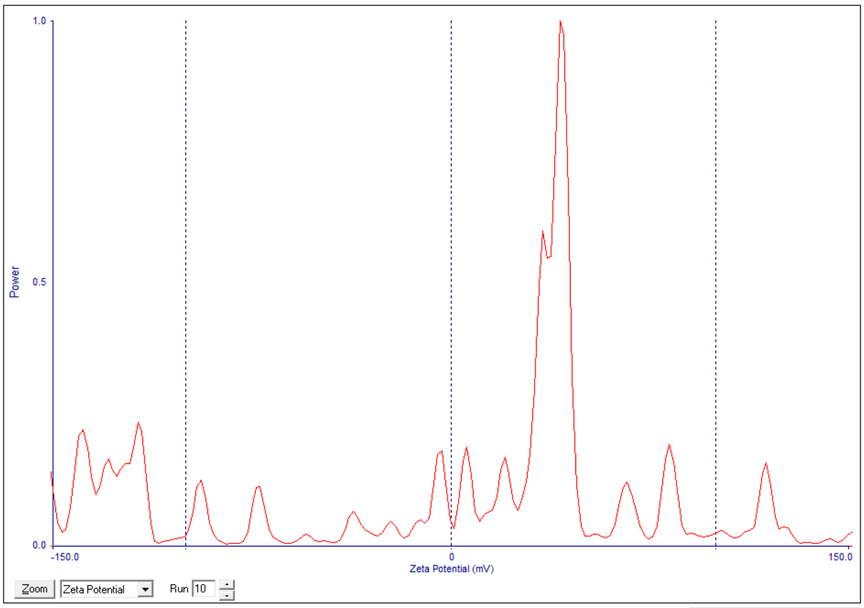

Zeta Potential

Run	Zeta Potential (mV)	Half Width (mV)
1	34.74	3.02
2	50.29	3.27
3	47.75	2.84
4	66.64	2.67
5	37.96	2.67
6	55.81	7.26
7	42.88	2.88
8	20.69	2.92
9	-19.35	2.78
10	41.14	5.92
Mean	44.21	3.72
Std. Error	4.38	0.56

Measurement Parameters:

Conductance	= 1147 µS
Current	= 6.31 mA
Electric Field	= 14.87 V/cm
Sample Count Rate	= 441 kcps
Ref. Count Rate	= 1151 kcps

Ag/14pl nAg/nSurf = 4 (Run 10)

Measurement Completed

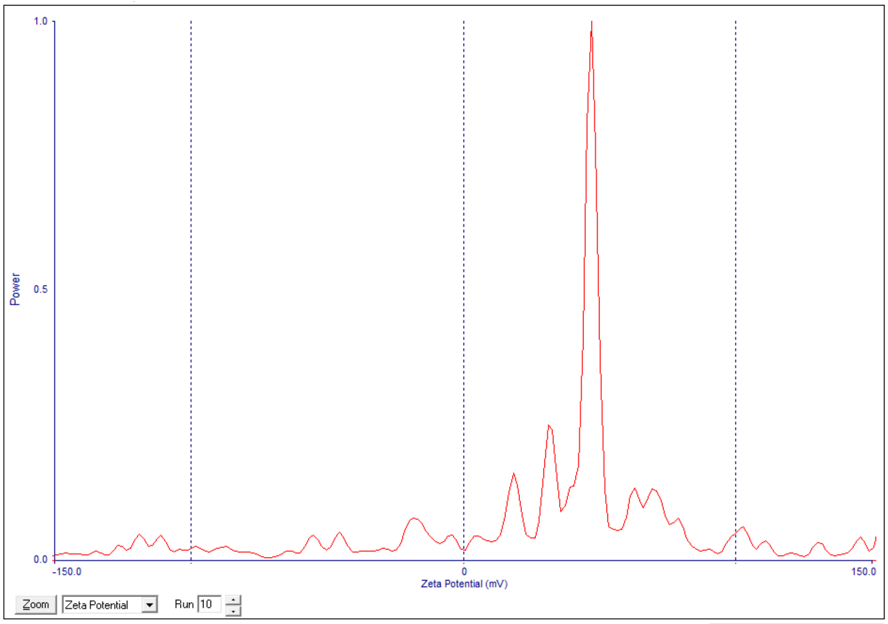

Zeta Potential

Run	Zeta Potential (mV)	Half Width (mV)
1	47.77	3.07
2	60.76	6.61
3	46.27	2.89
4	63.43	5.40
5	57.08	3.21
6	59.99	3.17
7	39.17	3.40
8	50.59	2.68
9	39.95	2.89
10	47.05	2.75
Mean	51.21	3.61
Std. Error	2.74	0.42

Measurement Parameters:

Conductance	= 1037 µS
Current	= 5.72 mA
Electric Field	= 14.91 V/cm
Sample Count Rate	= 485 kcps
Ref. Count Rate	= 974 kcps

Ag/14pl nAg/nSurf = 2.7 (Run 10)

Measurement Completed

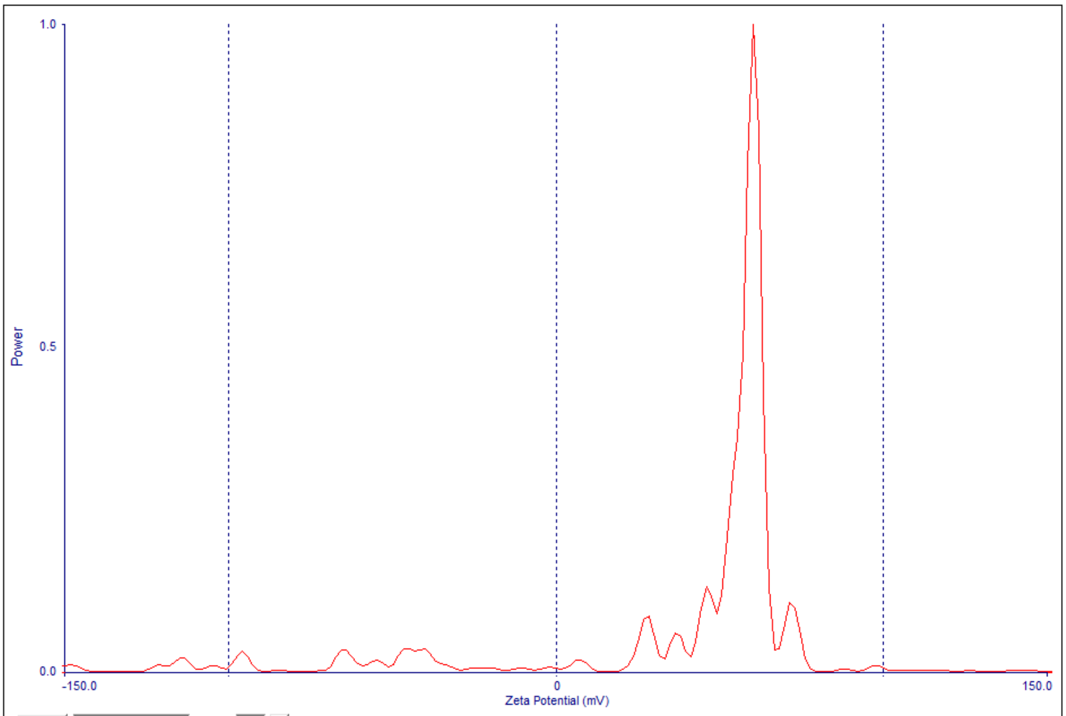

Zeta Potential

Run	Zeta Potential (mV)	Half Width (mV)
1	57.07	3.26
2	54.98	3.04
3	50.11	3.75
4	52.58	2.69
5	54.81	3.97
6	56.83	2.76
7	47.26	2.67
8	56.92	3.80
9	51.60	2.75
10	60.26	3.02
Mean	54.24	3.17
Std. Error	1.22	0.16

Measurement Parameters:

Conductance = 831 µS
Current = 4.60 mA
Electric Field = 15.08 V/cm
Sample Count Rate = 391 kcps
Ref. Count Rate = 916 kcps

Ag/14pl nAg/nSurf = 2 (Run 10)

Measurement Completed

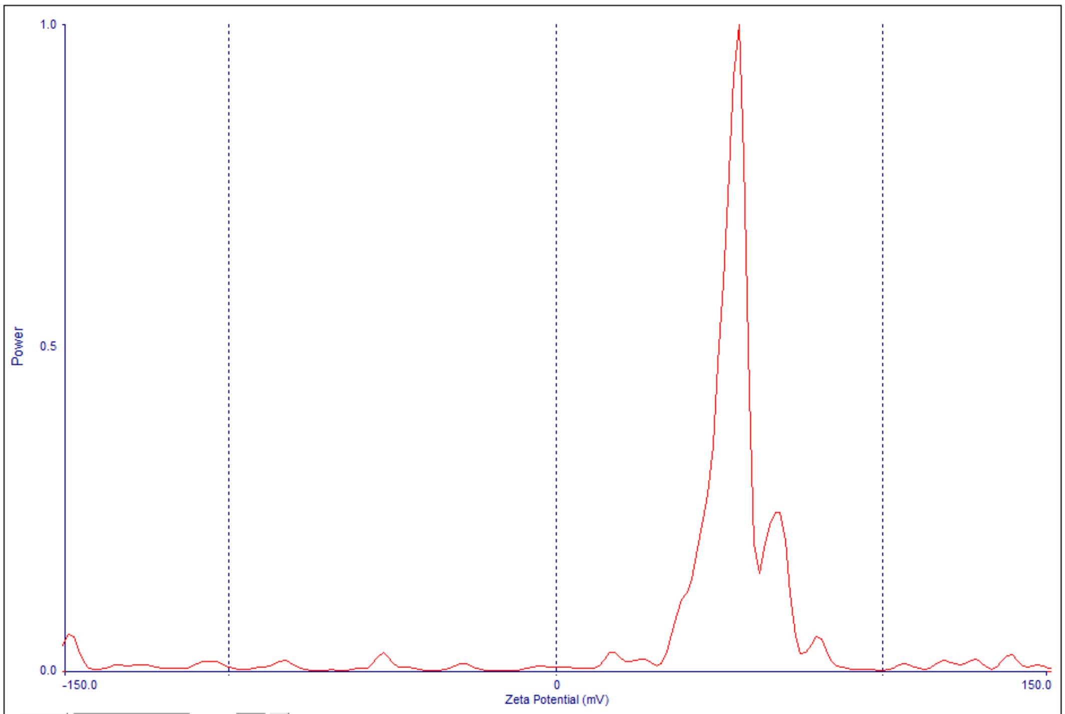

Zeta Potential

Run	Zeta Potential (mV)	Half Width (mV)
1	64.62	2.84
2	52.84	4.50
3	61.96	4.96
4	52.86	3.34
5	50.74	2.70
6	51.32	3.17
7	51.32	3.27
8	41.83	2.70
9	51.69	3.24
10	55.96	4.43
Mean	53.52	3.52
Std. Error	2.00	0.26

Measurement Parameters:

Conductance = 753 µS
Current = 4.18 mA
Electric Field = 15.27 V/cm
Sample Count Rate = 319 kcps
Ref. Count Rate = 897 kcps

Ag/15pl

Ag/15pl nAg/nSurf = 8 (Run 10)

Measurement Completed

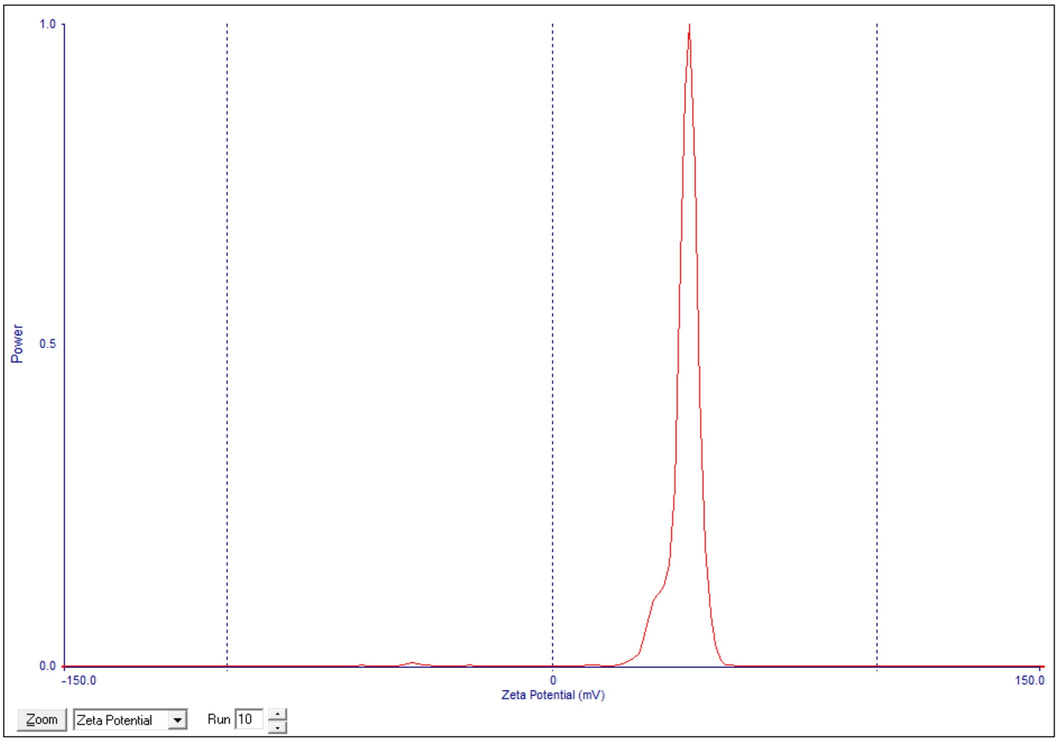

Zeta Potential

Run	Zeta Potential (mV)	Half Width (mV)
1	36.29	2.76
2	18.20	2.68
3	37.19	2.78
4	36.16	2.80
5	37.16	2.82
6	33.05	3.03
7	34.63	3.87
8	31.57	2.97
9	30.95	2.95
10	42.39	3.16
Mean	33.76	2.98
Std. Error	2.02	0.11

Measurement Parameters:

Conductance = 917 µS
Current = 5.06 mA
Electric Field = 15.01 V/cm
Sample Count Rate = 375 kcps
Ref. Count Rate = 1049 kcps

Ag/15pl nAg/nSurf = 4 (Run 10)

Measurement Completed

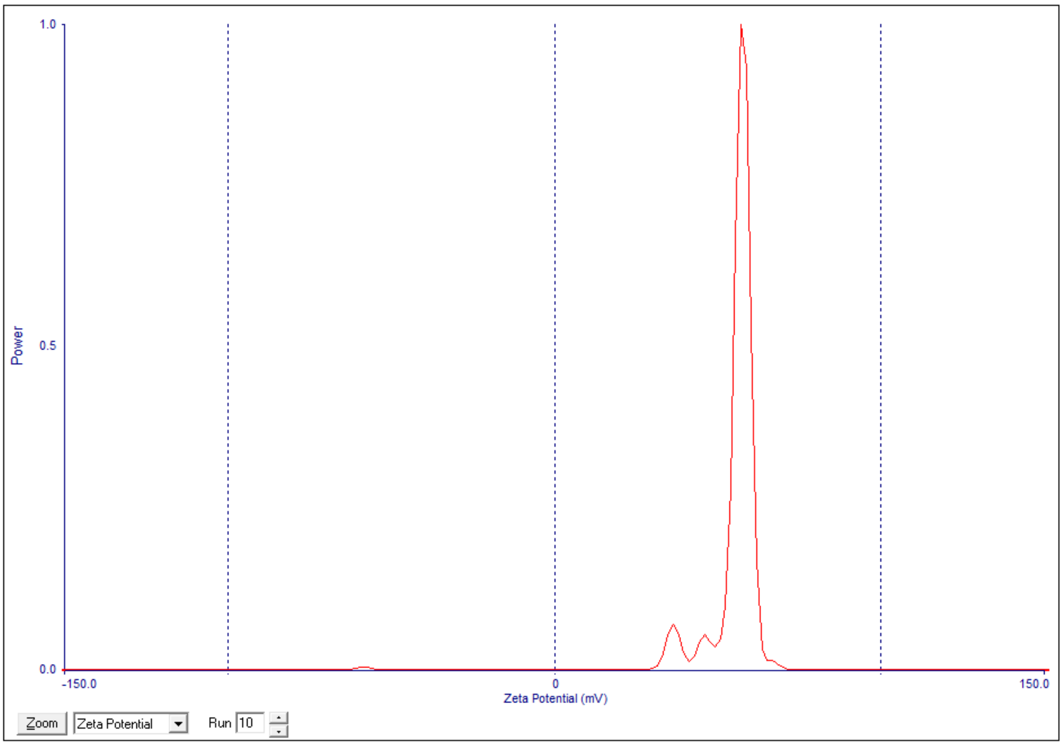

Zeta Potential

Run	Zeta Potential (mV)	Half Width (mV)
1	32.79	2.85
2	37.51	2.92
3	39.86	3.04
4	40.01	2.97
5	44.28	7.38
6	47.47	3.55
7	46.03	3.22
8	39.76	4.19
9	43.02	2.87
10	57.15	2.79
Mean	42.79	3.58
Std. Error	2.09	0.44

Measurement Parameters:

Conductance = 1075 µS
Current = 5.92 mA
Electric Field = 14.86 V/cm
Sample Count Rate = 312 kcps
Ref. Count Rate = 896 kcps

Ag/15pl nAg/nSurf = 2.7 (Run 10)

Measurement Completed

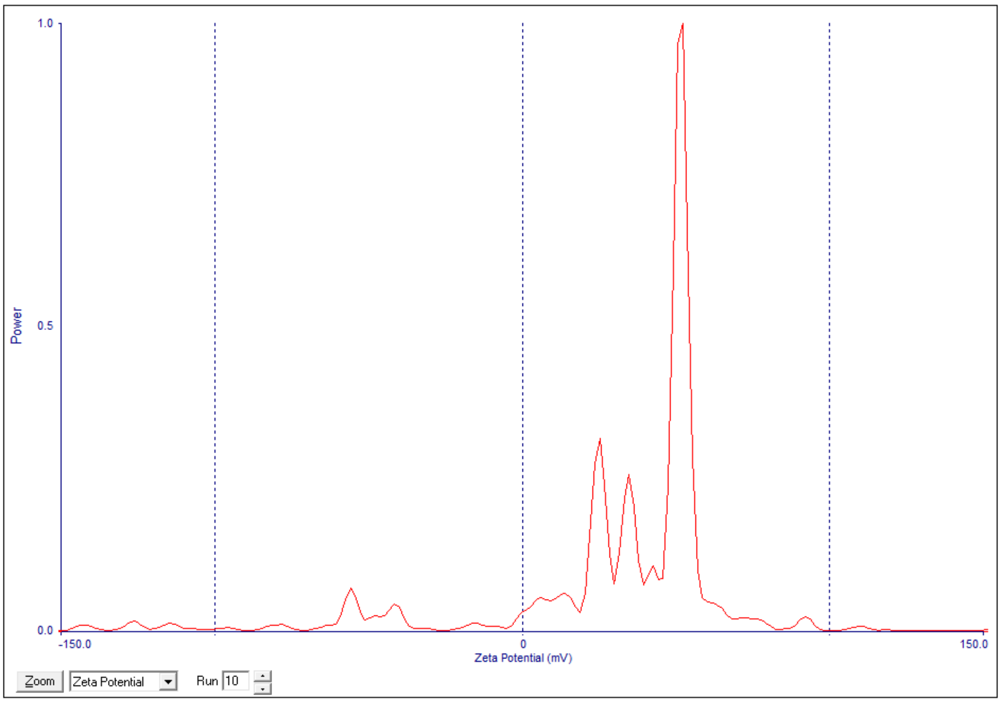

Zeta Potential

Run	Zeta Potential (mV)	Half Width (mV)
1	41.15	3.27
2	53.80	5.08
3	49.93	3.19
4	52.17	3.05
5	64.98	3.37
6	59.88	2.76
7	55.28	3.44
8	56.98	3.76
9	53.22	3.19
10	52.26	2.94
Mean	53.97	3.40
Std. Error	1.98	0.21

Measurement Parameters:
Conductance = 1015 µS
Current = 5.60 mA
Electric Field = 14.93 V/cm
Sample Count Rate = 408 kcps
Ref. Count Rate = 950 kcps

Ag/15pl nAg/nSurf = 2 (Run 10)

Measurement Completed

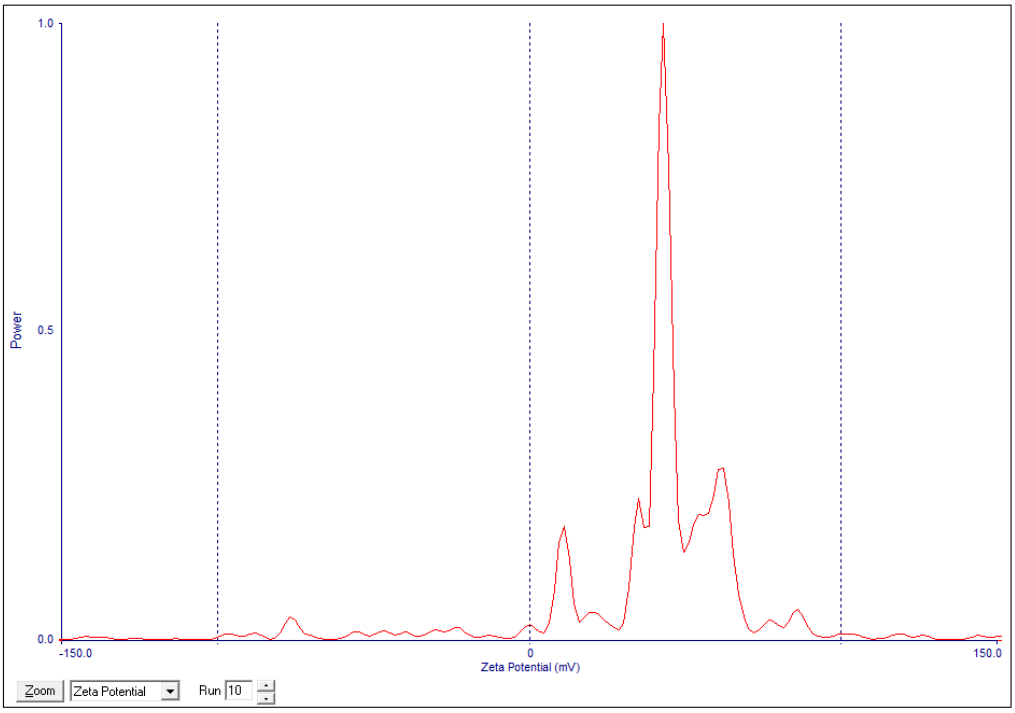

Zeta Potential

Run	Zeta Potential (mV)	Half Width (mV)
1	51.75	3.85
2	64.53	3.70
3	65.82	3.34
4	62.23	3.38
5	55.34	2.67
6	66.79	2.80
7	57.07	2.80
8	55.42	3.10
9	58.34	3.68
10	43.15	2.84
Mean	58.04	3.22
Std. Error	2.29	0.14

Measurement Parameters:
Conductance = 632 µS
Current = 3.53 mA
Electric Field = 15.08 V/cm
Sample Count Rate = 440 kcps
Ref. Count Rate = 937 kcps

Ag16/pl

Ag/16pl nAg/nSurf = 8 (Run 10)
Measurement Completed

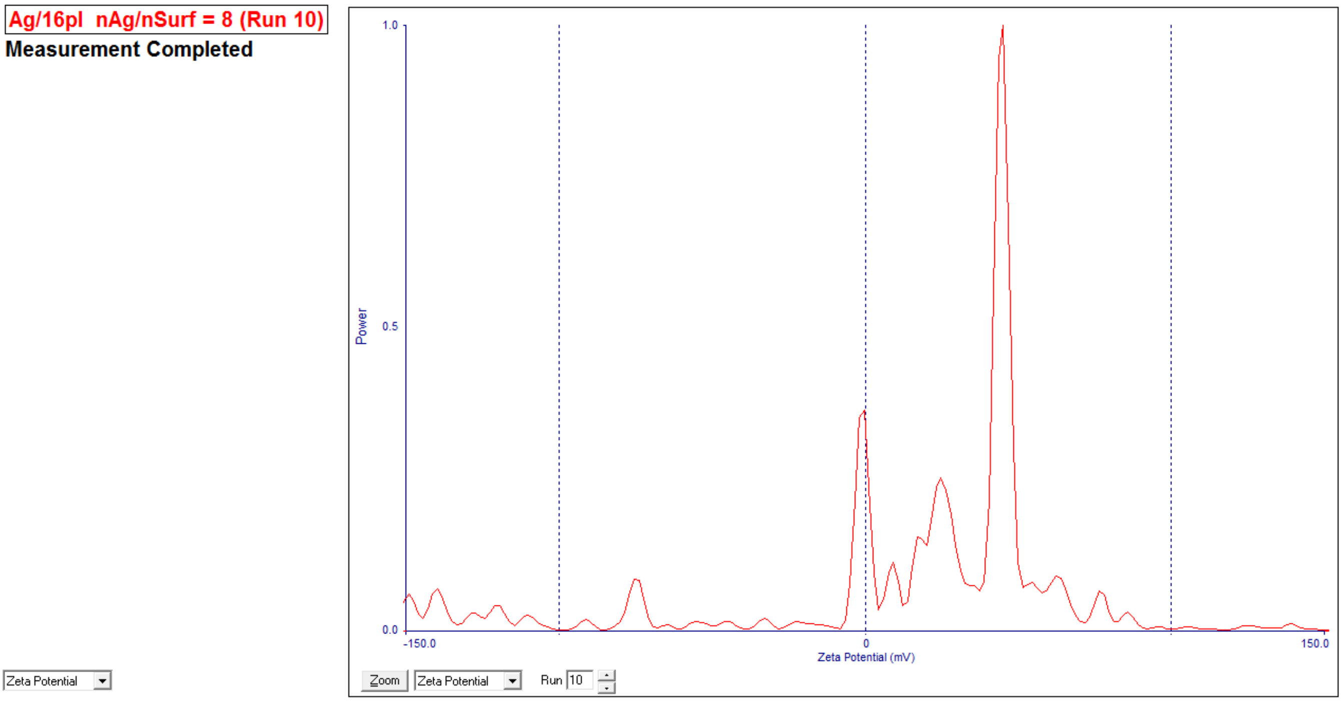

Run	Zeta Potential (mV)	Half Width (mV)
1	32.92	3.88
2	23.63	3.08
3	48.33	2.77
4	31.23	3.06
5	53.43	3.17
6	45.86	3.80
7	46.99	2.83
8	43.97	3.72
9	35.98	6.16
10	45.21	2.85
Mean	40.76	3.53
Std. Error	2.95	0.32

Measurement Parameters:
Conductance = 1206 µ S
Current = 6.63 mA
Electric Field = 14.92 V/cm
Sample Count Rate = 422 kcps
Ref. Count Rate = 1140 kcps

Ag/16pl nAg/nSurf = 4 (Run 10)
Measurement Completed

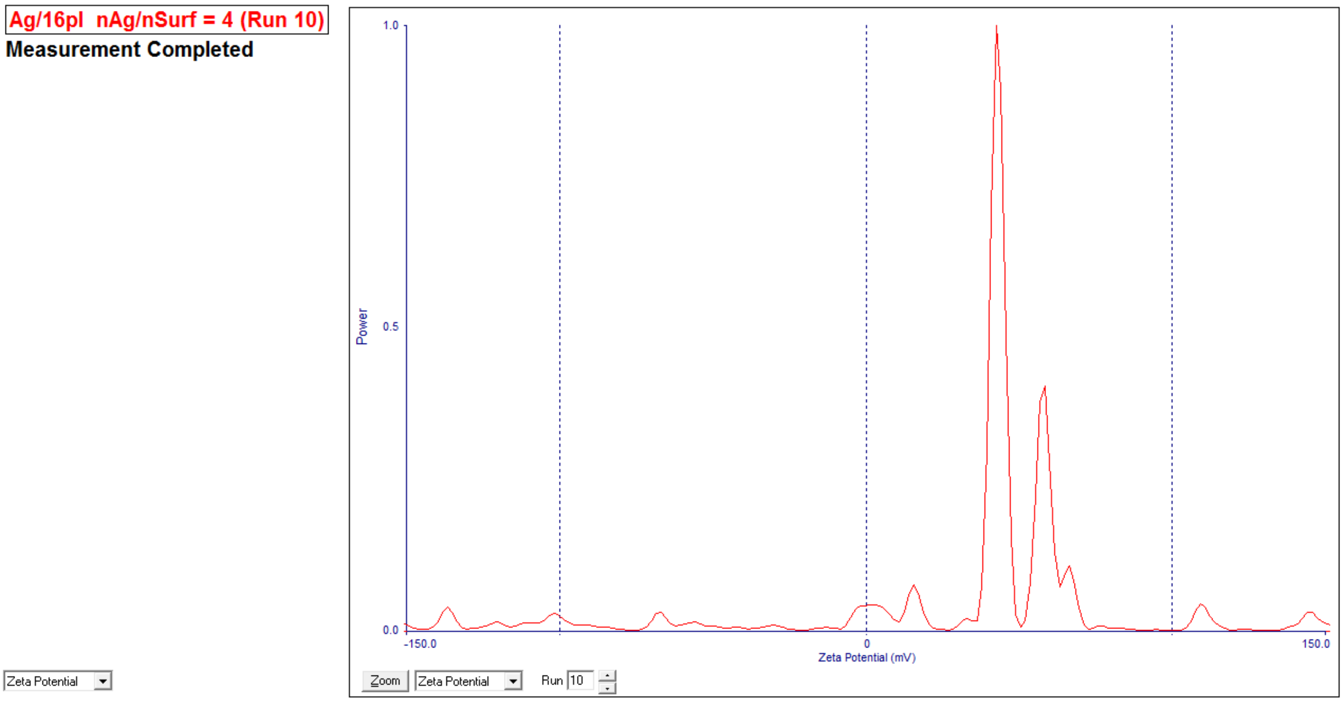

Run	Zeta Potential (mV)	Half Width (mV)
1	46.81	2.83
2	36.33	2.88
3	51.98	3.20
4	45.97	3.00
5	68.15	2.90
6	63.42	2.65
7	63.13	2.93
8	45.95	2.92
9	61.36	3.29
10	42.59	2.75
Mean	52.57	2.94
Std. Error	3.39	0.06

Measurement Parameters:
Conductance = 1360 µ S
Current = 7.46 mA
Electric Field = 14.86 V/cm
Sample Count Rate = 395 kcps
Ref. Count Rate = 972 kcps

Ag/16pl nAg/nSurf = 2.7 (Run 10)

Measurement Completed

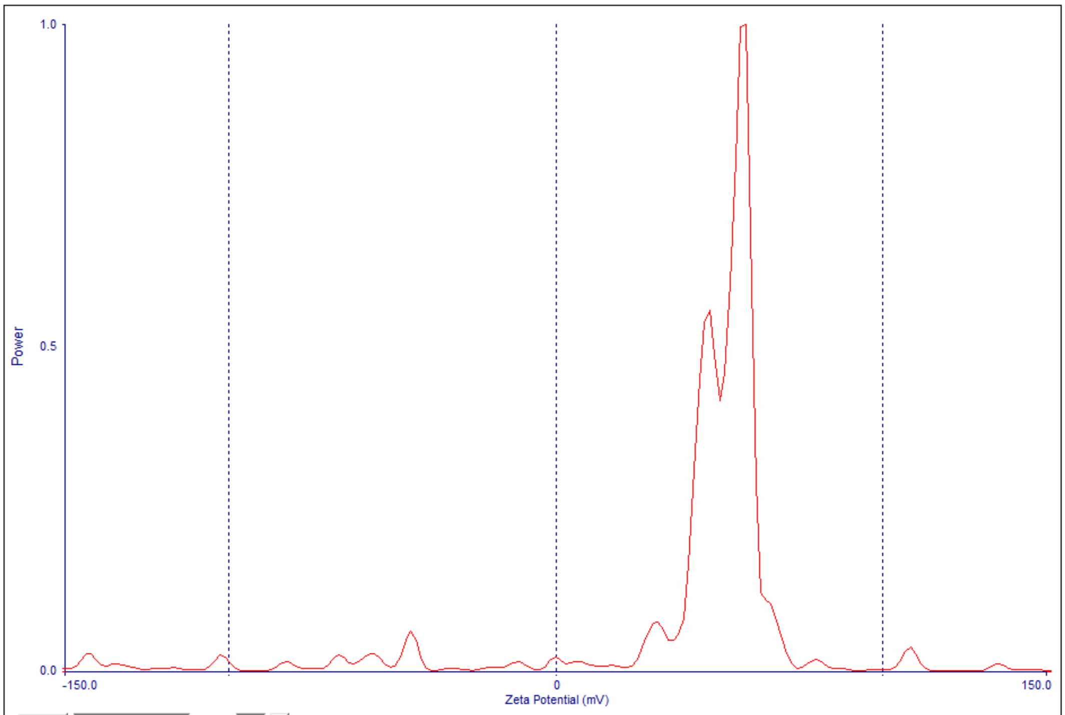

Zeta Potential | Zoom | Zeta Potential | Run 10

Run	Zeta Potential (mV)	Half Width (mV)
1	55.28	2.95
2	59.73	3.06
3	35.57	2.77
4	-44.54	3.20
5	-122.16	3.38
6	50.10	3.01
7	48.08	3.74
8	61.42	4.77
9	47.14	3.21
10	58.04	4.08
Mean	54.26	3.55
Std. Error	2.20	0.26

Measurement Parameters:	
Conductance	= 990 µS
Current	= 5.46 mA
Electric Field	= 15.02 V/cm
Sample Count Rate	= 360 kcps
Ref. Count Rate	= 909 kcps

Ag/16pl nAg/nSurf = 2 (Run 10)

Measurement Completed

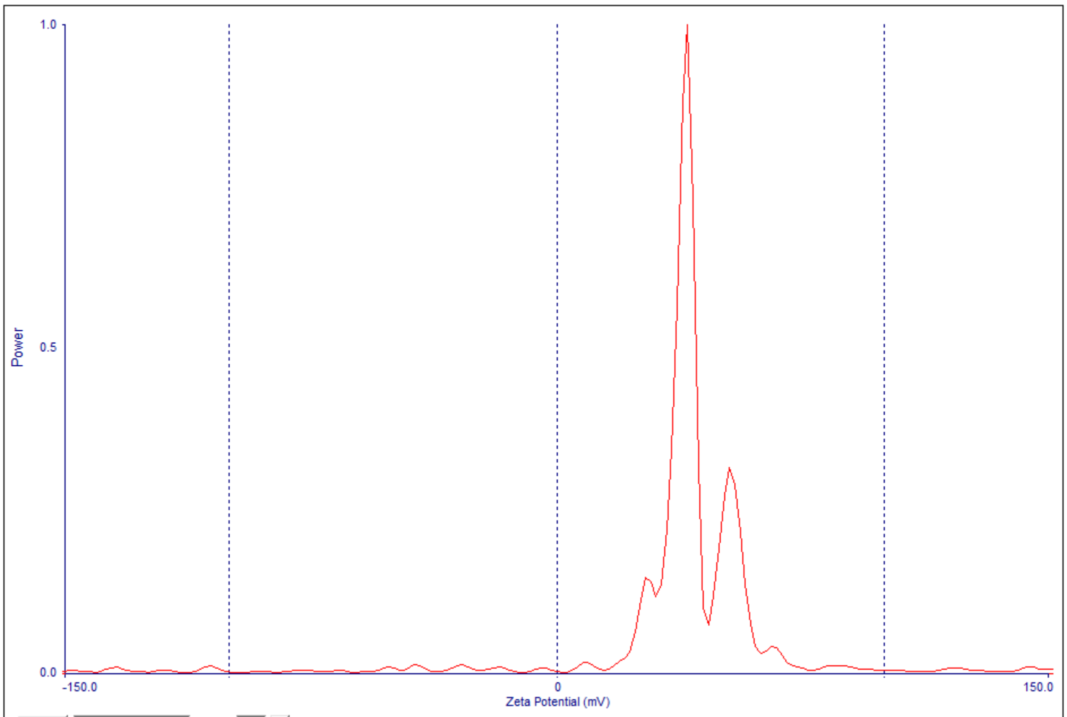

Zeta Potential | Zoom | Zeta Potential | Run 10

Run	Zeta Potential (mV)	Half Width (mV)
1	47.22	2.78
2	48.71	5.48
3	55.26	4.60
4	46.64	2.98
5	42.53	2.87
6	51.99	3.36
7	48.78	5.37
8	43.14	3.40
9	42.25	4.91
10	39.96	3.08
Mean	46.65	3.88
Std. Error	1.51	0.34

Measurement Parameters:	
Conductance	= 672 µS
Current	= 3.74 mA
Electric Field	= 15.00 V/cm
Sample Count Rate	= 351 kcps
Ref. Count Rate	= 947 kcps

Ag/17pl

Ag/17pl nAg/nSurf = 8 (Run 10)

Measurement Completed

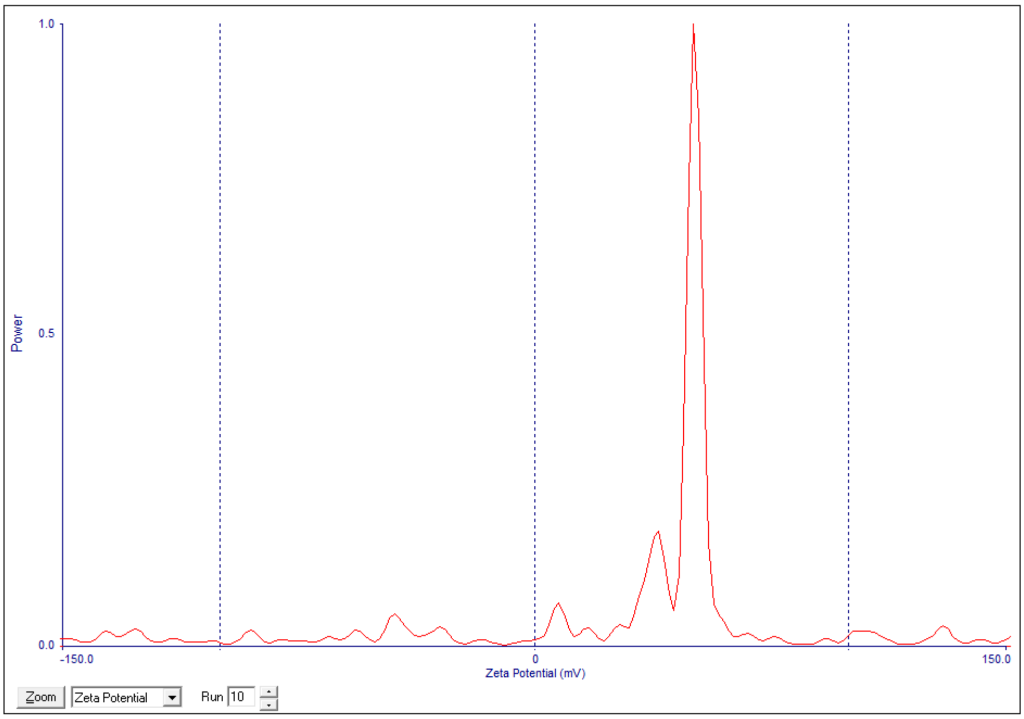

Zeta Potential

Run	Zeta Potential (mV)	Half Width (mV)
1	40.40	4.33
2	58.64	2.85
3	45.87	3.32
4	54.58	3.11
5	40.91	2.72
6	59.43	2.78
7	44.24	2.71
8	52.03	4.10
9	46.76	3.99
10	50.74	2.78
Mean	49.36	3.27
Std. Error	2.16	0.20

Measurement Parameters:

Conductance	= 920 µ S
Current	= 5.08 mA
Electric Field	= 14.93 V/cm
Sample Count Rate	= 251 kcps
Ref. Count Rate	= 1266 kcps

Ag/17pl nAg/nSurf = 4 (Run 10)

Measurement Completed

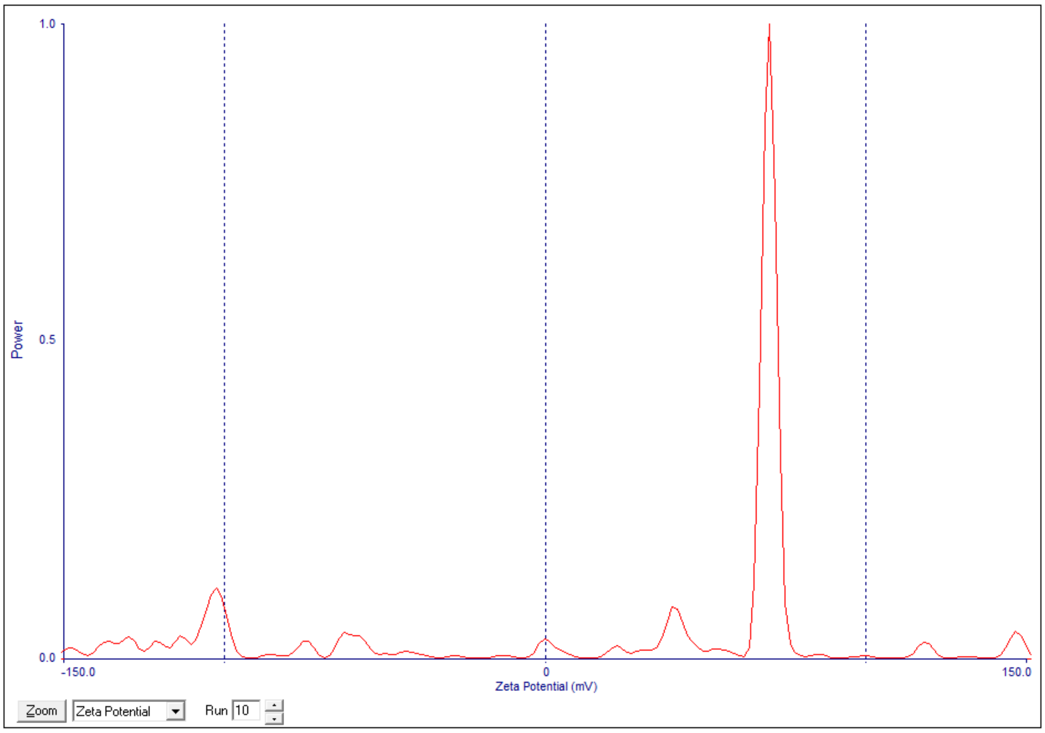

Zeta Potential

Run	Zeta Potential (mV)	Half Width (mV)
1	65.20	2.71
2	45.93	3.00
3	53.73	3.20
4	58.87	3.11
5	57.04	5.43
6	-165.80	1.88
7	56.63	4.39
8	49.13	2.63
9	-124.47	2.82
10	70.03	2.70
Mean	57.07	3.40
Std. Error	2.79	0.35

Measurement Parameters:

Conductance	= 998 µ S
Current	= 5.50 mA
Electric Field	= 14.92 V/cm
Sample Count Rate	= 492 kcps
Ref. Count Rate	= 1086 kcps

Ag/17pl nAg/nSurf = 2.7 (Run 10)

Measurement Completed

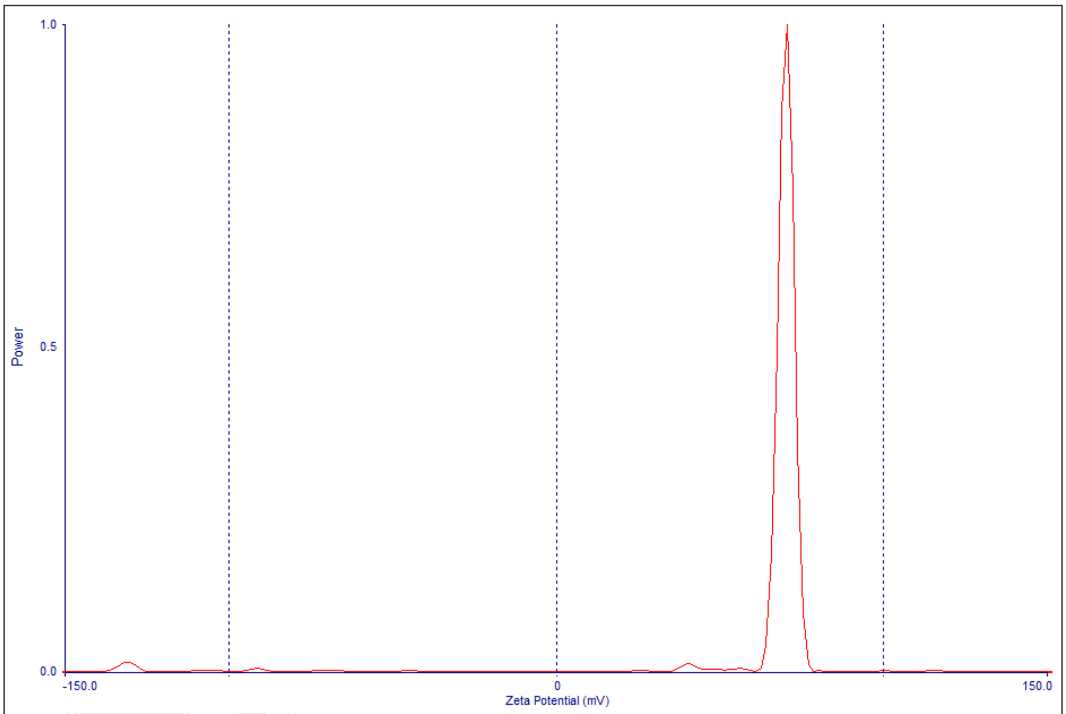

Run	Zeta Potential (mV)	Half Width (mV)
1	43.05	2.86
2	50.82	6.15
3	50.31	2.97
4	46.63	2.80
5	54.49	3.14
6	60.56	3.05
7	61.21	2.60
8	62.74	2.96
9	55.73	3.18
10	70.59	2.88
Mean	55.61	3.26
Std. Error	2.63	0.33

Measurement Parameters:

Conductance = 804 µS
Current = 4.46 mA
Electric Field = 14.84 V/cm
Sample Count Rate = 399 kcps
Ref. Count Rate = 900 kcps

Ag/17pl nAg/nSurf = 2 (Run 10)

Measurement Completed

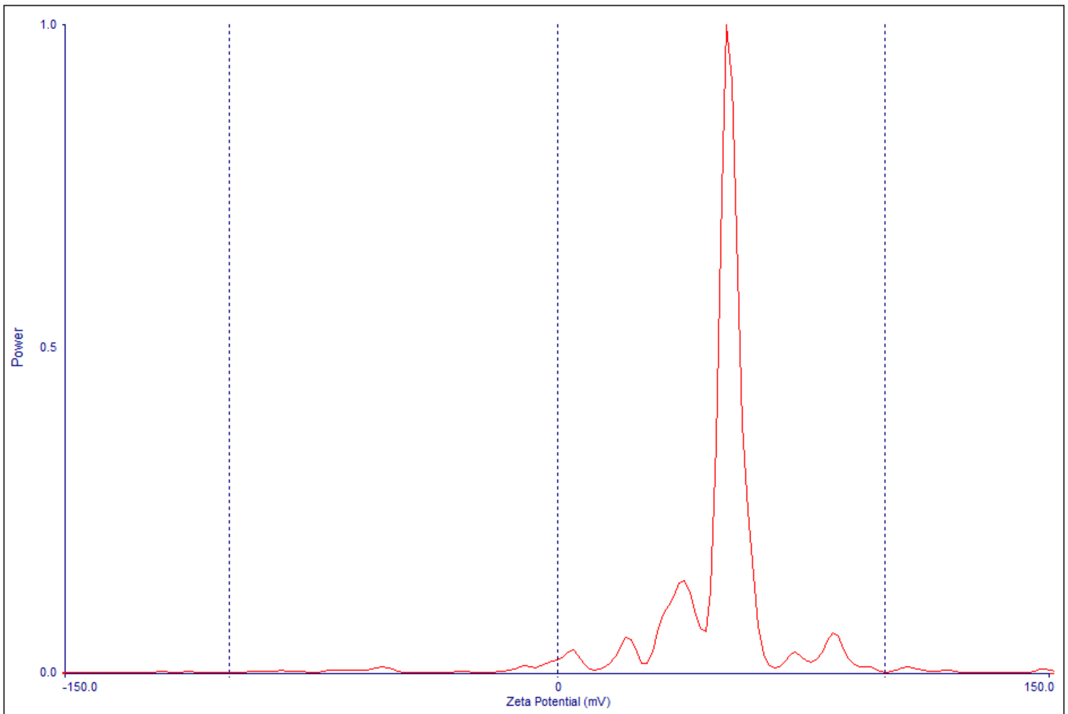

Run	Zeta Potential (mV)	Half Width (mV)
1	57.28	7.36
2	81.68	2.61
3	47.92	3.47
4	51.66	4.38
5	50.67	2.99
6	62.78	3.04
7	55.83	4.07
8	61.10	5.93
9	55.85	2.90
10	51.74	3.33
Mean	57.65	4.01
Std. Error	3.05	0.48

Measurement Parameters:

Conductance = 730 µS
Current = 4.06 mA
Electric Field = 14.86 V/cm
Sample Count Rate = 318 kcps
Ref. Count Rate = 886 kcps

Ag/18pl

Ag/18pl nAg/nSurf = 8 (Run 10)

Measurement Completed

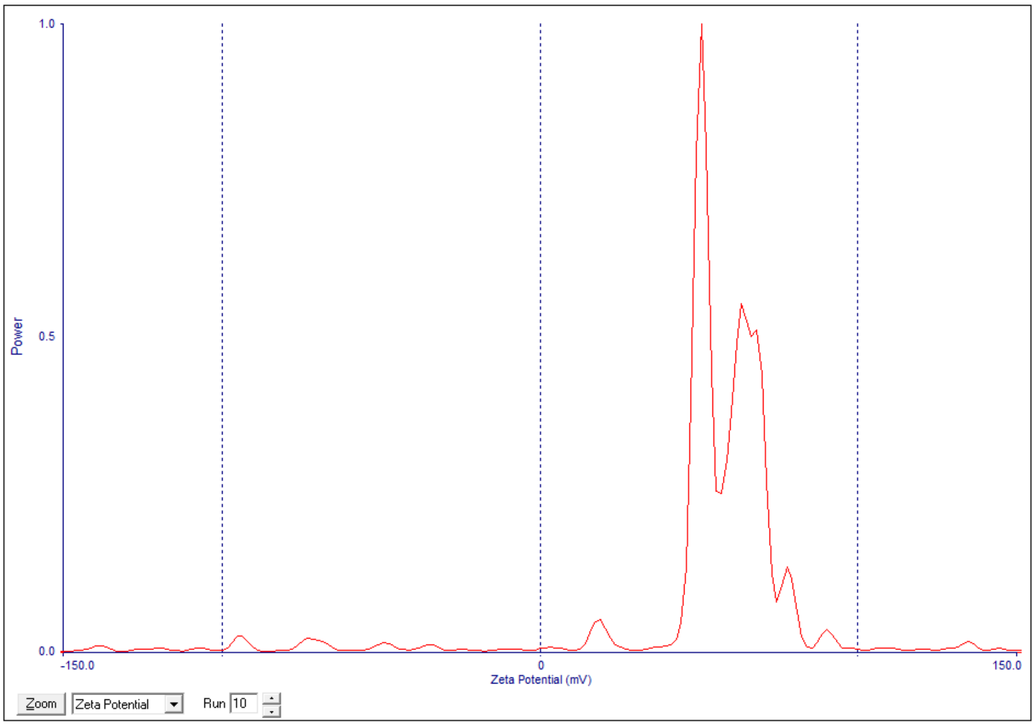

Zeta Potential

Run	Zeta Potential (mV)	Half Width (mV)
1	61.40	3.14
2	66.90	3.05
3	35.99	6.50
4	45.51	2.56
5	69.75	3.00
6	70.90	3.22
7	64.01	2.71
8	68.66	3.05
9	62.99	3.24
10	50.76	2.84
Mean	62.32	2.98
Std. Error	2.91	0.08

Measurement Parameters:

Conductance = 1207 µ S
Current = 6.63 mA
Electric Field = 15.03 V/cm
Sample Count Rate = 452 kcps
Ref. Count Rate = 906 kcps

Ag/18pl nAg/nSurf = 4 (Run 10)

Measurement Completed

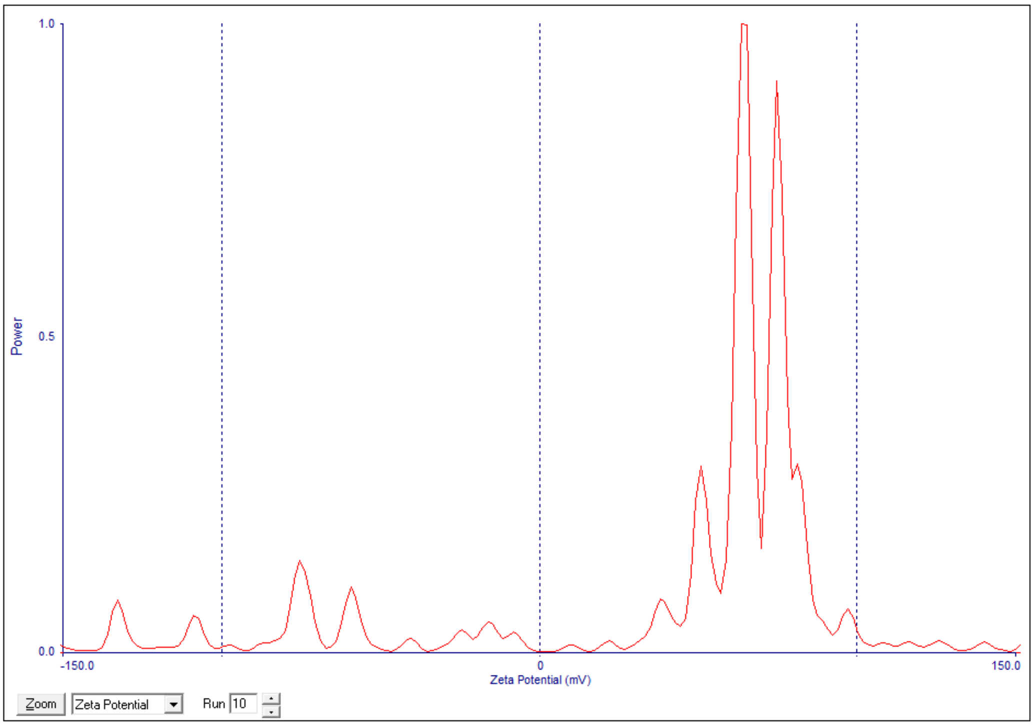

Zeta Potential

Run	Zeta Potential (mV)	Half Width (mV)
1	71.83	2.79
2	76.46	6.07
3	76.04	3.56
4	55.73	2.71
5	64.67	2.86
6	65.59	3.16
7	69.71	4.04
8	75.16	2.63
9	72.64	5.87
10	63.66	3.21
Mean	69.15	3.69
Std. Error	2.11	0.40

Measurement Parameters:

Conductance = 1113 µ S
Current = 6.12 mA
Electric Field = 14.90 V/cm
Sample Count Rate = 190 kcps
Ref. Count Rate = 876 kcps

Ag/18pl nAg/nSurf = 2.7 (Run 10)

Measurement Completed

Zeta Potential

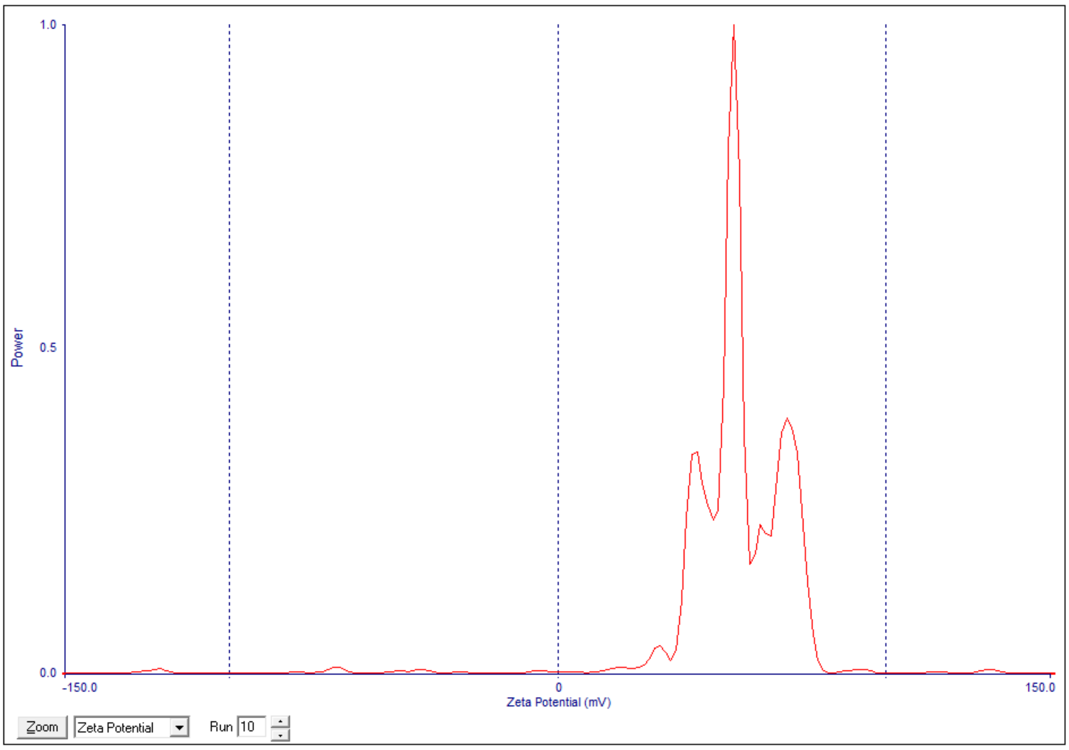

Run	Zeta Potential (mV)	Half Width (mV)
1	66.58	3.41
2	49.34	3.03
3	73.27	3.29
4	75.01	2.86
5	76.89	2.88
6	68.64	5.15
7	63.98	2.76
8	53.55	3.04
9	67.32	3.28
10	53.71	2.82
Mean	66.55	3.28
Std. Error	2.81	0.25

Measurement Parameters:

Conductance = 931 µ S
Current = 5.14 mA
Electric Field = 14.90 V/cm
Sample Count Rate = 202 kcps
Ref. Count Rate = 895 kcps

Ag/18pl nAg/nSurf = 2 (Run 10)

Measurement Completed

Zeta Potential

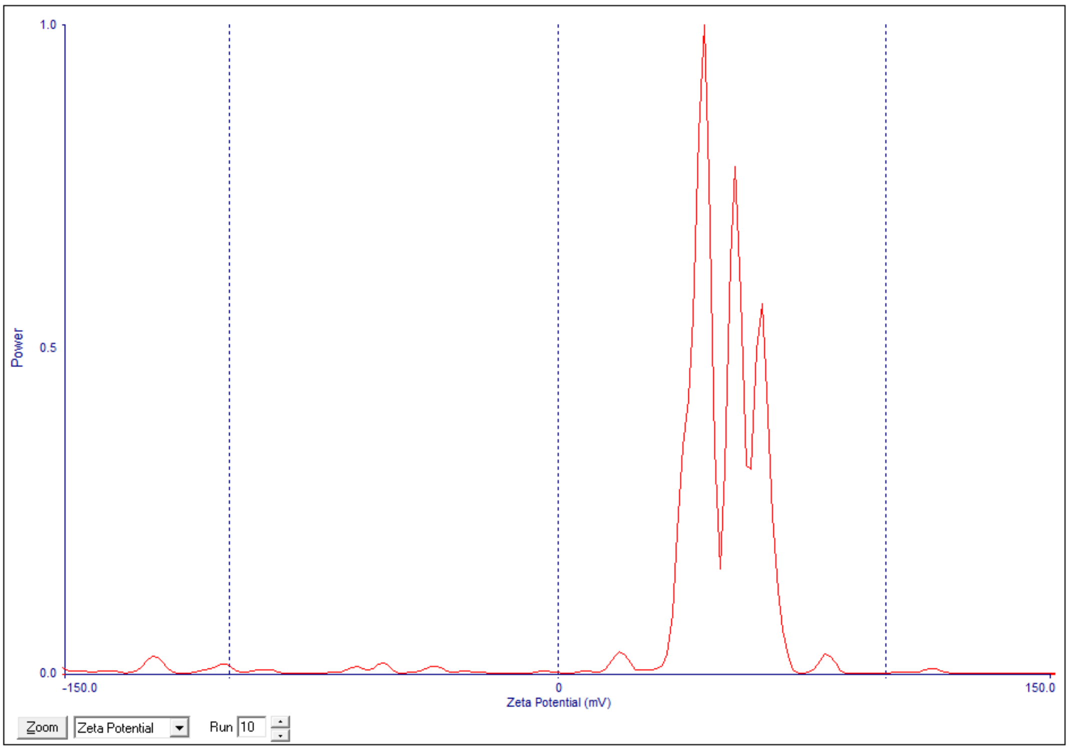

Run	Zeta Potential (mV)	Half Width (mV)
1	68.01	3.05
2	64.01	2.89
3	51.95	2.96
4	61.71	4.22
5	79.43	2.77
6	44.71	5.04
7	62.38	3.03
8	50.96	5.87
9	62.53	3.43
10	44.58	3.22
Mean	62.62	3.53
Std. Error	3.17	0.37

Measurement Parameters:

Conductance = 635 µ S
Current = 3.54 mA
Electric Field = 14.98 V/cm
Sample Count Rate = 336 kcps
Ref. Count Rate = 880 kcps

Image of the used Zeta potential analyser:

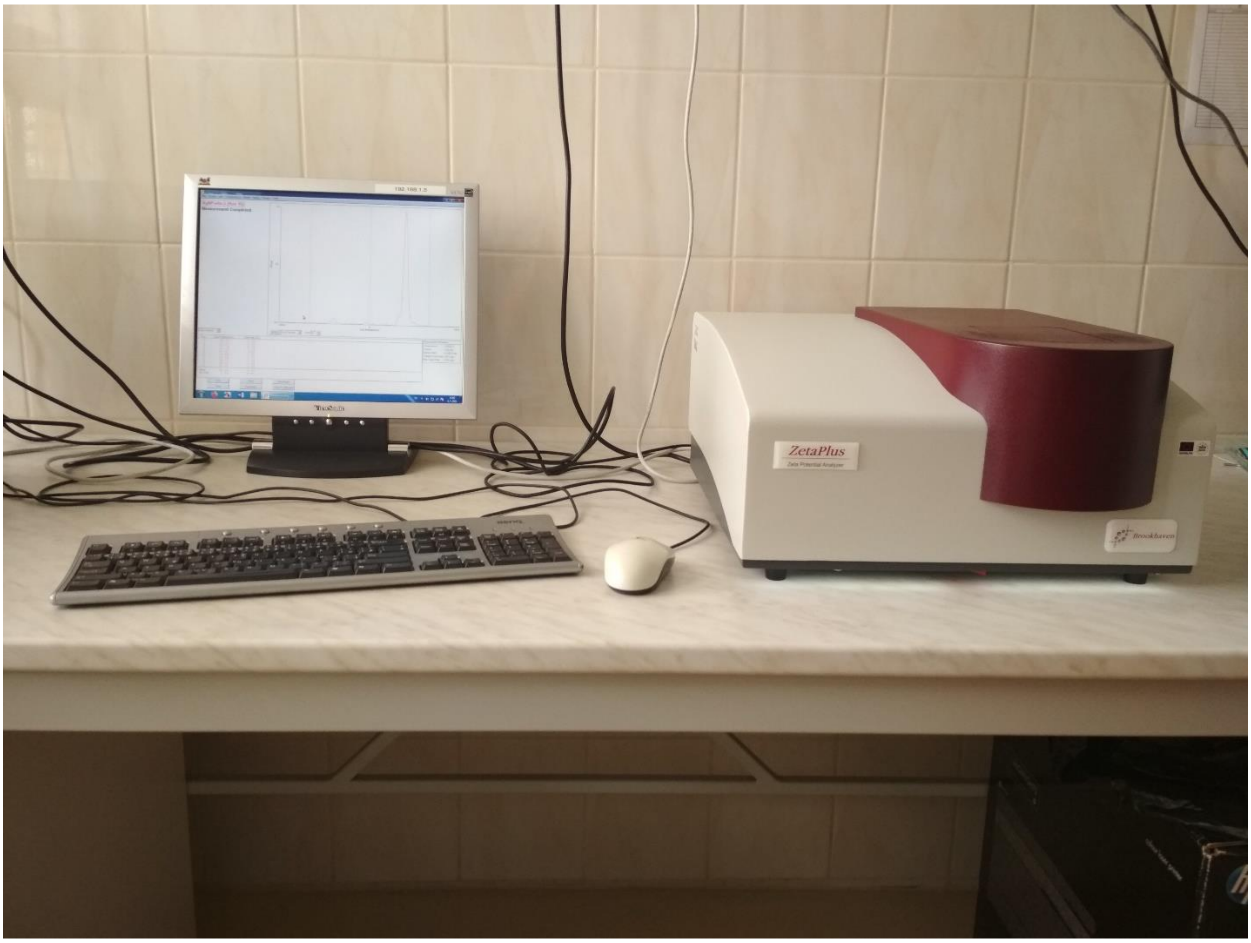

3. SEM high resolution images
Ag/14pl

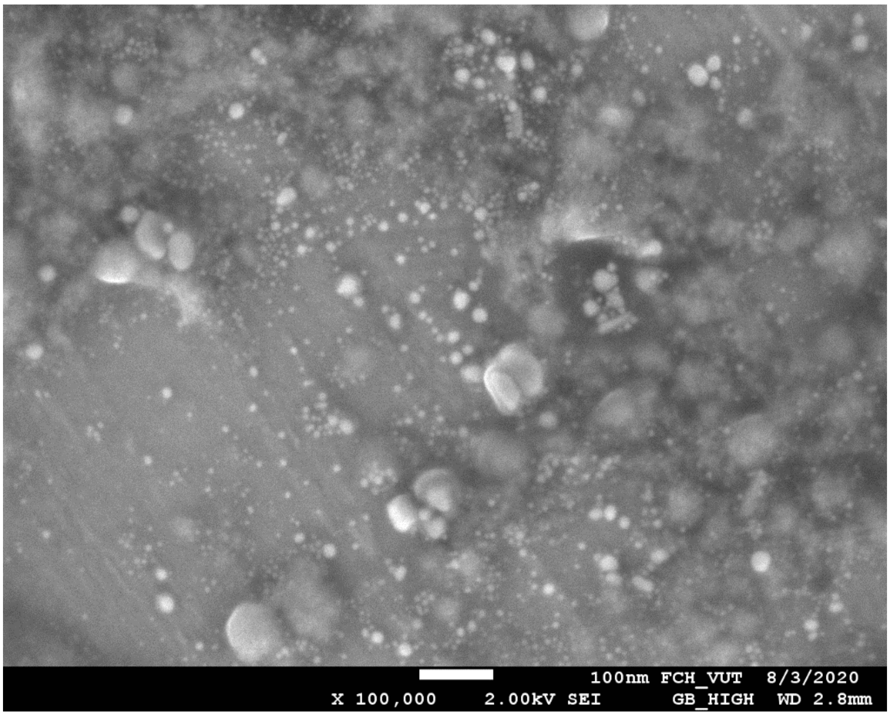

Ag/15pl

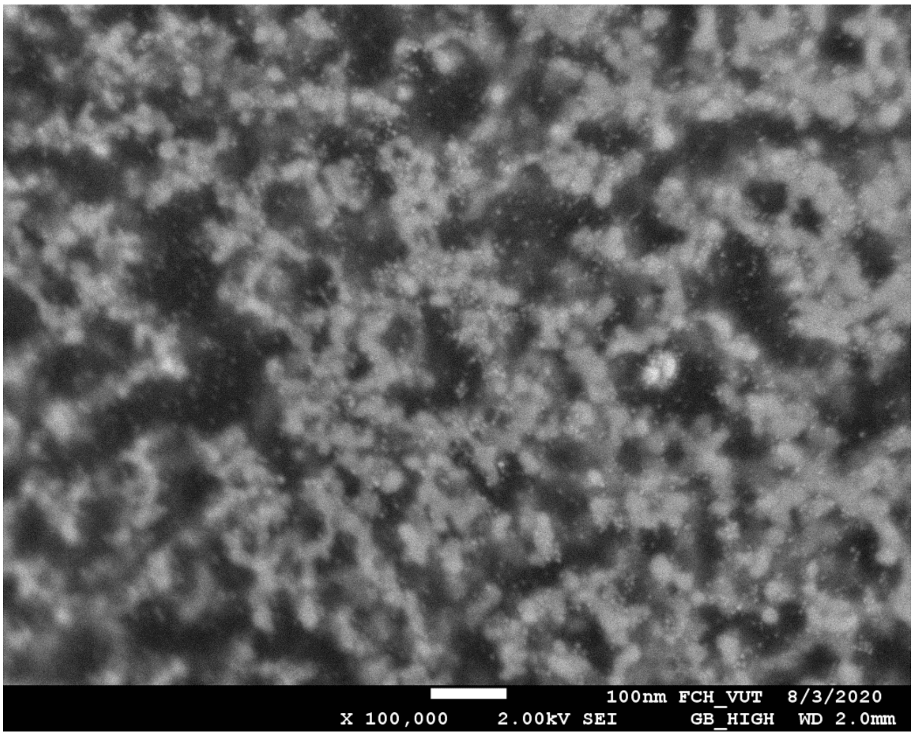

Ag/16pl

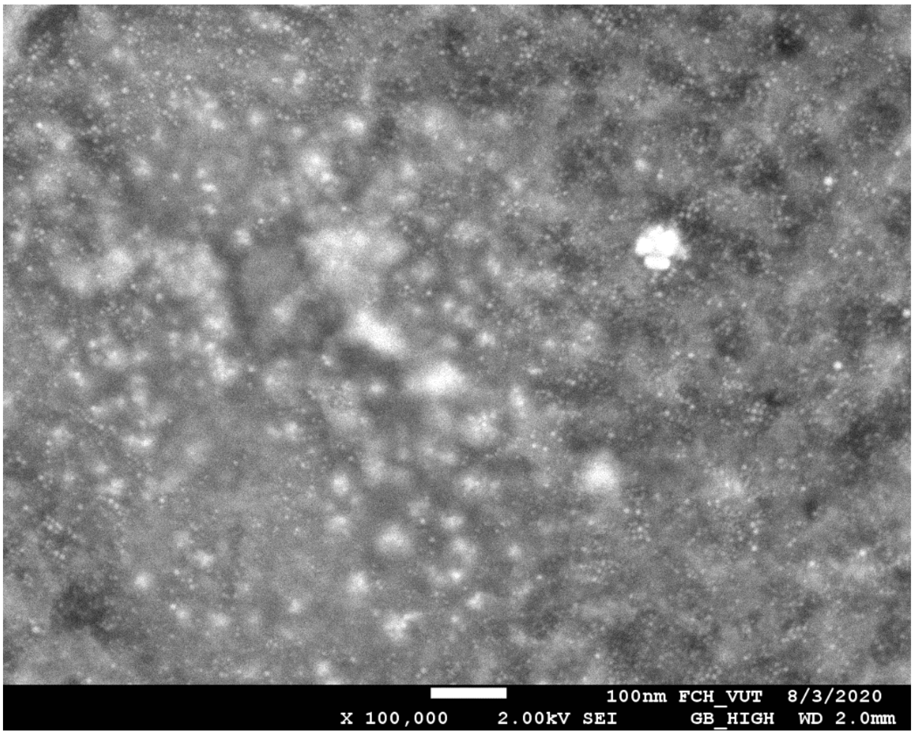

Ag/17pl

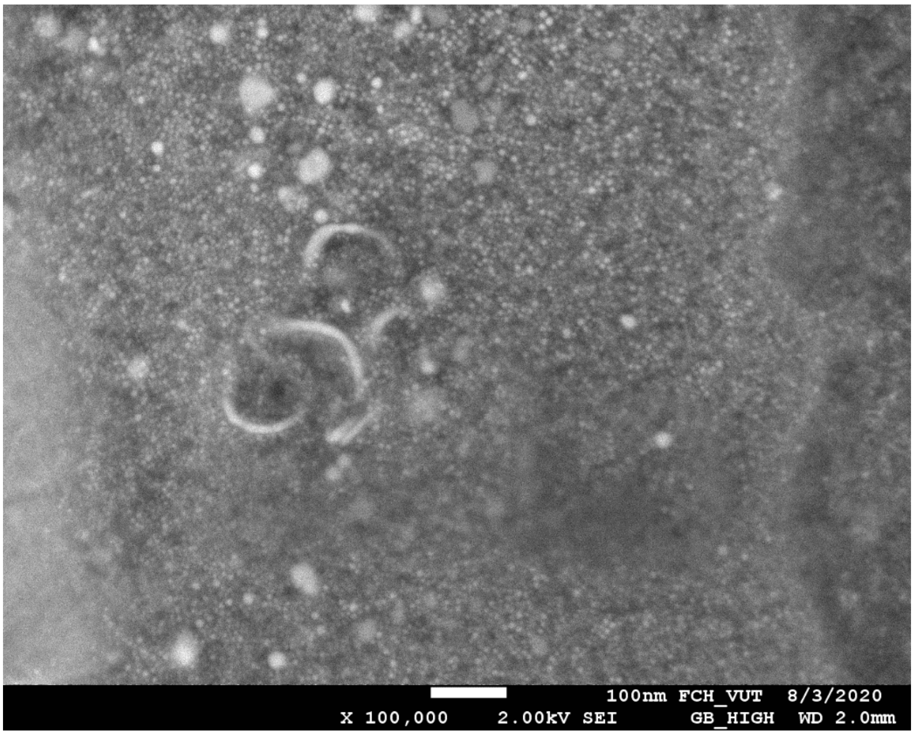

Ag/18pl

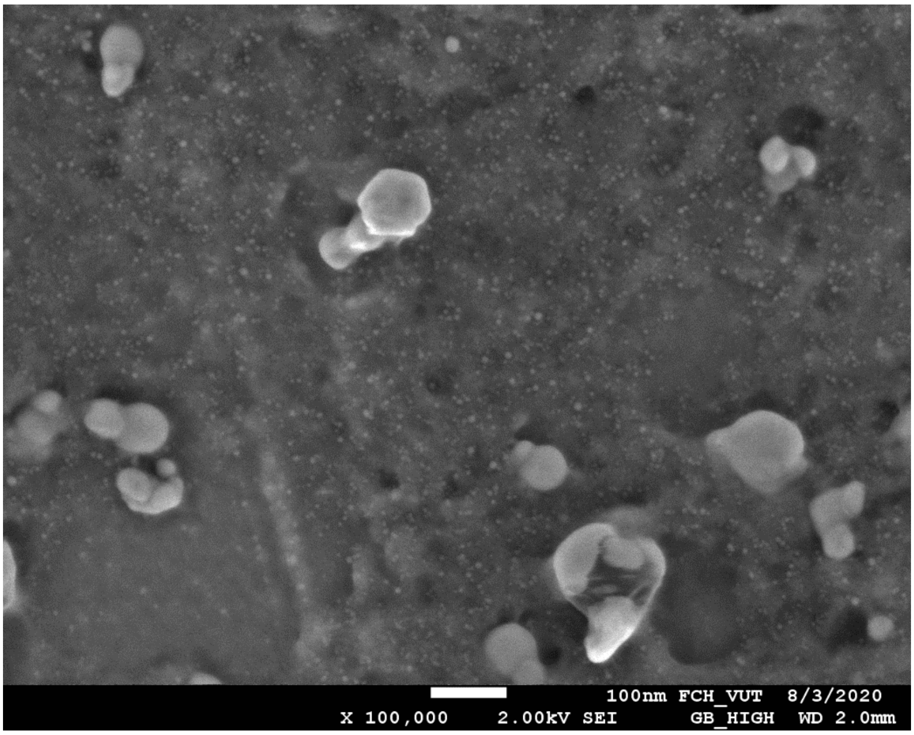

Supplement: Supplementary file 1 [file nanomaterials-11-01883-s001.zip › nanomaterials-1266020-supplementary.pdf]
